# USP33 Promotes Lung Adenocarcinoma Brain Metastasis by Inhibiting the K48-Linked Ubiquitination and Degradation of S100A9 and Facilitating Vimentin Secretion

**DOI:** 10.7150/ijbs.127941

**Published:** 2026-05-29

**Authors:** Zihao Wang, Mengqi Chang, Yuekun Wang, Yang Qu, Wenbo Wu, Jiahui Liu, Xiaopeng Guo, Yidong Zhou, Wenbin Ma, Yu Wang

**Affiliations:** 1 Department of Breast Surgery, Peking Union Medical College Hospital, Chinese Academy of Medical Sciences and Peking Union Medical College, Beijing, China.; 2 Department of Neurosurgery, Peking Union Medical College Hospital, Chinese Academy of Medical Sciences and Peking Union Medical College, Beijing, China.; 3 Medical Research Center, State Key Laboratory of Complex Severe and Rare Diseases, Peking Union Medical College Hospital, Chinese Academy of Medical Sciences and Peking Union Medical College, Beijing, China.

**Keywords:** LUAD, brain metastasis, S100A9, USP33, extracellular vimentin, blood-brain barrier

## Abstract

Brain metastasis has a deterministic influence on the evolutionary trajectory of lung adenocarcinoma (LUAD). Given the profound clinical importance of LUAD brain metastases and the formidable obstacles that hinder their precise prediction, novel therapeutic targets are urgently needed. Notably, a substantial fraction of lung carcinoma cells in brain metastatic foci demonstrates marked S100A9 overexpression. However, whether these cells represent the primary drivers of brain metastasis requires further elucidation. Coculture of S100A9-overexpressing LUAD cells with human brain microvascular endothelial cells (HBMECs) markedly suppressed the expression of the tight junction components ZO-1, Occludin, and Claudin-5 within the endothelial barrier. These cocultured HBMECs also exhibited compromised angiogenic potential, elevated levels of reactive oxygen species, and increased endothelial-to-mesenchymal transition. Additionally, these cells displayed attenuated proliferative and migratory capacities and mitochondrial membrane potential depolarization along with increased autophagy characterized by increased lysosomal acidification. Conversely, LUAD cells with elevated S100A9 expression exhibited increased proliferative, migratory, and invasive capacities and increased tumorigenicity and potential for brain metastasis. In LUAD cell lines, USP33 stabilizes S100A9 via K48-linked deubiquitination, which promotes the expression and extracellular secretion of vimentin. LUAD cells reduce blood‒brain barrier integrity, increase permeability, and disrupt the intra- and extracellular functions of HBMECs, thus promoting parenchymal infiltration and the establishment of metastatic lesions.

## Introduction

Lung adenocarcinoma (LUAD), the predominant histological subtype of non-small cell lung cancer (NSCLC), displays a marked predilection for metastatic propagation, with specific tropism toward the central nervous system and brain 1. Epidemiological estimates indicate that 38% of patients with advanced LUAD ultimately develop brain metastases, constituting the predominant site of distant recurrence 2. This severe complication predominantly underlies the very low five-year survival rate (< 20%) of these patients 3, and after the diagnosis of brain metastases, the median survival is typically less than 12 months 4. Although diverse malignancies exhibit the potential for cerebral metastasis, the prevalence of such metastases remains high in lung cancer patients 5. Notably, LUAD-derived intracranial metastases exhibit a distinct topographic predilection for the frontal lobe 6. Leptomeningeal metastasis, a critical and prevalent sequela, is pathologically characterized by disruption of the blood-brain barrier (BBB) and a resultant increase in vascular permeability 7. Such metastases exhibit a robust association with rapid declines in cognitive and neurological function, a reduced capacity to perform normal daily activities, and a profound increase in mortality rates 8. Existing therapeutic modalities, such as surgical resection, radiotherapy, chemotherapy, and immunotherapy, confer limited clinical benefit 9. Accordingly, elucidating the pathophysiological and molecular mechanisms underlying LUAD-associated brain metastasis and identifying novel therapeutic targets are urgent research directions.

Recent innovations in omics technologies and clinical science have led to the identification of specific biomarkers associated with LUAD brain metastases. While circulating tumor-derived markers, including CEA, CA125, CA19-9, CYFRA21-1, Cathepsin F, and Fibulin-1, have been extensively investigated, their utility in early metastasis detection and prognosis remains constrained 10. Specifically, suboptimal sensitivity and specificity necessitate their use in combination and further rigorous clinical validation.

In a prior study that performed single-cell sequencing of matched primary tumor (PT) and brain metastasis specimens, we characterized a distinct malignant epithelial subpopulation, named brain metastasis-associated epithelial cells, and established that S100A9 is its defining molecular signature using computational algorithms 11. Similarly, in high-grade serous carcinoma (HGSC), a malignancy characterized by early peritoneal dissemination, S100A9-positive tumor cell subsets exhibit preferential enrichment at metastatic sites, demonstrate increased glycolytic flux, and correlate with poor survival outcomes 12. Bone marrow metastasis and disseminated intravascular coagulation (DIC) are common complications of aggressive gastric neoplasms. In this context, the upregulation of S100A8/A9 synergistically orchestrates DIC progression with the activation of the VEGF, PDGF, FGF, and NOTCH signaling cascades 13.

S100A9, an EF-hand calcium-binding protein in the S100 family, exhibits heterogeneous and context-specific expression profiles across diverse malignancies 14. In the context of pancreatic carcinoma pulmonary metastasis, S100A9 orchestrates neutrophil recruitment and neutrophil extracellular trap formation, thereby fostering metastatic colonization 15. In colorectal cancer, S100A9 drives hepatic metastasis through early lymphatic dissemination 16 and amplifies tumor cell proliferation, invasion, and metastatic competence via the CXCL5/CXCL2 signaling axis 17. In addition to its effects on tumor cells, S100A9 modulates the tumor microenvironment (TME) via intricate crosstalk with immune cells, fibroblasts, and endothelial cells. Furthermore, S100A9 overexpression in ER+/HER2- breast cancer *in vitro* coculture systems increases natural killer cell cytotoxicity 18. S100A9 promotes the progression of ovarian cancer through the mobilization of neutrophils, myeloid dendritic cells, CD4+ T cells, and mast cells within inflammatory niches 19. Highly aggressive small cell lung cancer models exhibit upregulated S100A9 expression, which potentiates tumor cell migration and invasion through the Akt and GSK3α/β/Snail transduction pathways, inhibits autophagy and myeloid-derived suppressor cell recruitment, and increases CD8+ T-cell infiltration, thereby synergistically reprogramming the TME and promoting metastasis 20. In the context of glioma, S100A9 is released into the TME, triggering microglial M2 polarization and increasing TGFβ1 secretion while compromising CD8+ T-cell effector function, thereby establishing a protumorigenic niche 21. CD74 potently modulates S100A9 expression, culminating in increased inflammatory cytokine production by fibroblasts and the promotion of pancreatic ductal adenocarcinoma progression 22. Upon engagement with ligands such as Toll-like receptor 4 and the receptor for advanced glycation end products, S100A9 activates myeloid-derived suppressor cells and promotes melanoma progression 23. Pharmacological blockade of S100A9 with tasquinimod inhibits myeloma cell proliferation, increases T-cell effector function, alleviates immunosuppression, and impedes multiple myeloma progression 24. S100A9 also activates NF-κB signaling to facilitate cutaneous squamous cell carcinoma tumorigenesis 25. S100A9 is a hallmark of highly activated macrophages and interacts with endothelial cells to promote angiogenesis and facilitate tumor growth 26. In the context of oral squamous cell carcinoma, S100A9 promotes angiogenesis and is upregulated in neutrophils via the Chemerin/MEK/ERK signaling axis 27. Despite these insights, the precise role of S100A9 in the pathogenesis of LUAD remains insufficiently elucidated. This investigation focuses on the intratumoral expression of S100A9, rather than that in myeloid-derived populations, and elucidates its contribution to brain metastasis, specifically its compromise of blood‒brain barrier integrity during leptomeningeal dissemination. Thus, it is imperative to identify S100A9-interacting proteins and elucidate the associated signaling cascades.

Ubiquitination, a reversible posttranslational modification, is rigorously orchestrated by the antagonistic interplay between ubiquitin ligases and deubiquitinating enzymes 28. By performing coimmunoprecipitation integrated with mass spectrometry, we characterized ubiquitin-specific peptidase 33 (USP33) as a previously unidentified deubiquitinase of S100A9. Despite the established role of USP33 in diverse malignancies, its functional significance within the context of brain metastases remains insufficiently elucidated. Prior investigations have demonstrated that USP33 cleaves K27- and K48-linked polyubiquitin chains from Chromobox Homolog 2, thereby stabilizing the CBX2 protein and facilitating the progression of ovarian cancer 29. In pancreatic cancer, USP33 mediates the deubiquitination of transforming growth factor-beta receptor 2, which results in its accumulation at the cellular membrane, sustained TGF-β signaling, the activation of Zinc Finger E-Box Binding Homeobox 1, and the establishment of a prometastatic feedback loop 30. The results of the current study elucidate that USP33 increases the stability of S100A9 via the selective hydrolysis of K48-linked polyubiquitin chains, thereby compromising the blood‒brain barrier and potentiating brain metastasis in the context of LUAD.

Vimentin, a type III intermediate filament constituent, plays a pivotal role in oncobiology. Its aberrant overexpression in epithelial cells promotes epithelial‒mesenchymal transition, thereby promoting tumor proliferation, migration, and chemotherapeutic resistance 31. Tumor-associated endothelial cells frequently exhibit aberrant overexpression and secretion of vimentin via unconventional secretory pathways 32. Transcriptomic profiling in this investigation revealed that vimentin expression increased in S100A9-overexpressing LUAD cells. Consequently, the functional interplay between S100A9 and vimentin in orchestrating the phenotypic plasticity of human brain microvascular endothelial cells (HBMECs) merits further comprehensive elucidation.

HBMECs, the fundamental components of the BBB, typically exhibit increased expression of claudin-5 and tight junction protein 1 in pathological contexts 33, 34; conversely, their angiogenic potential is attenuated 35. Reactive oxygen species (ROS) serve as potent cellular stressors, damaging to vital biomolecules and compromising HBMEC function 36. The attenuated proliferative and migratory capacities of and mitochondrial dysfunction in HBMECs potentiate the disruption of BBB integrity 37. Moreover, increased autophagic flux in these cells attenuates immune surveillance at the BBB and promotes disease progression 38. The present investigation investigates whether S100A9-mediated intercellular crosstalk between LUAD cells and HBMECs initiates BBB compromise and modulates metastatic progression.

Through the utilization of a LUAD-cerebrovascular endothelial cell coculture system, we recapitulated S100A9-induced BBB disruption *in vitro*. Key objectives of this study included determining whether USP33-mediated K48-linked deubiquitination stabilizes S100A9, increases vimentin expression and secretion, and consequently promotes S100A9-high LUAD cells to compromise endothelial function and precipitate brain metastasis. Understanding these processes will substantially advance our knowledge of the pathophysiology underlying LUAD brain metastasis and reveal potential therapeutic targets for intervention.

## Methods

### Cell Lines, Cell Culture, and Reagents

The human LUAD cell line A549 (CL-0016, Procell) was cultured in Ham's F-12K medium supplemented with 10% fetal bovine serum (FBS) and 1% penicillin‒streptomycin (P/S) to maintain optimal proliferation. The NCI-H1975 (CL-0298, Procell), PC-9 (CL-0668, Procell), and NCI-H1299 (CL-0165, Procell) cell lines were cultured in RPMI-1640 medium supplemented with identical concentrations of FBS and P/S. Additionally, NCI-H2009 cells were maintained in DMEM/F12 supplemented with 10% FBS and 1% P/S, whereas 293T cells (CL-0005, Procell) were cultured in standard DMEM supplemented with 10% FBS and 1% P/S. HBMECs (IM-H206, IMMOCELL) were cultivated in a proprietary endothelial cell growth medium optimized with growth factors. All the cell cultures were incubated at 37°C in a humidified atmosphere with 95% air and 5% CO₂ to maintain physiological homeostasis.

### Construction of Stable Cell Lines

To generate stable cell lines, A549 and NCI-H1975 cells at 50-60% confluence were transduced with lentiviral vectors (vector, S100A9-OE) at the calculated viral titers in serum-free medium to maximize infection efficiency. After 24 hours of transduction, the cells were thoroughly washed with phosphate-buffered saline, and the culture medium was replaced with fresh complete culture medium. Selection pressure was imposed with puromycin at 48 hours post-transduction and maintained for 4 weeks to eliminate nontransduced cells. Transduction efficiency and target gene expression were subsequently confirmed by Western blotting and quantitative PCR.

### Coculture of LUAD Cells and HBMECs

To establish a coculture system, the basolateral surface of a Transwell membrane insert (0.4 μm) was uniformly coated with 0.1% gelatin and incubated overnight at 4 °C to facilitate cellular adhesion. HBMECs were seeded into the upper chamber at a defined density of 2×10⁵ cells/cm², while complete growth medium was added to the lower chamber. The culture medium was replaced at 48-hour intervals, and the cells were maintained for 4 days to establish a confluent, functional monolayer. Following monolayer establishment, the medium was aspirated from both chambers. The HBMECs in the upper chamber were gently rinsed twice with prewarmed PBS to eliminate debris. Subsequently, LUAD cells in the logarithmic phase were harvested, washed, resuspended in serum-free coculture medium, and seeded into the lower chamber at a density of 5×10⁴ cells/cm². The coculture plates were incubated at 37 °C with 5% CO₂ for 48 hours for use in subsequent assays. All experiments were performed in triplicate to guarantee statistical reproducibility.

### CRISPR/Cas9-Mediated Gene Editing

Single-guide RNAs (sgRNAs) targeting specific exons of the S100A9 gene were designed with an online platform and selected on the basis of anticipated on-target efficiency. After reaching approximately 50% confluence, HEK293T cells were transfected, and after 24 hours, the transfection medium was replaced with fresh DMEM supplemented with 10% FBS. Viral supernatants were harvested 48 hours post-transfection, centrifuged at 3,000 × g for 10 minutes at 4°C to eliminate cellular debris, and subsequently filtered through a 0.45 µm membrane. Polybrene (8 µg/mL) was added to the filtrate to increase viral infectivity. Transduced HEK293T cells were subjected to selection with 1 µg/mL puromycin beginning 48 hours post-transfection. The knockout efficiency was rigorously validated via Western blotting and qPCR, with a nontargeting sgRNA used as the negative control.

### Reverse Transcription-Quantitative PCR (RT‒qPCR)

Total RNA was extracted with TRIzol reagent, followed by vigorous vortexing and centrifugation at 12,000 × g for 15 minutes at 4 °C to facilitate phase separation. RNA was precipitated with isopropanol for 10 minutes at room temperature and then pelleted via centrifugation at 12,000 × g for 15 minutes at 4 °C. The resulting RNA precipitate was washed with 75% ethanol to eliminate impurities, dried in air and finally resuspended in RNase-free water. The RNA concentration and purity were quantified utilizing a BioSpectrometer basic. Complementary DNA was synthesized with a Tiangen KR118-02 reverse transcription kit. RT‒qPCR was performed with SuperReal PreMix Plus (Tiangen FP205-02) on a StepOnePlus real-time PCR system. Glyceraldehyde-3-phosphate dehydrogenase (GAPDH) served as the endogenous control, and relative gene expression levels were calculated using the 2^-ΔΔCt^ method. Specific primer sequences are provided in **Table S1**.

### Enzyme-Linked Immunosorbent Assay (ELISA)

Following a 30-minute equilibration at ambient temperature, the ELISA plates were washed and dried. Standards and samples (100 µL each) were aliquoted into the wells and incubated at 37 °C for 90 minutes to facilitate antigen immobilization, followed by extensive washing. Subsequently, 100 µL of biotinylated anti-Vimentin antibody was added, and the samples were incubated at 37 °C for 60 minutes. Following aspiration of the solution, a horseradish peroxidase (HRP)-conjugated enzymatic complex was added for incubation at 37 °C for 30 minutes prior to subsequent washes. Then, a chromogenic substrate (100 µL) was added for incubation in the dark at 37 °C for 15 minutes to facilitate signal development. The enzymatic reaction was quenched by the addition of 50 µL of termination solution, and the optical density was measured at 450 nm within 5 minutes using a microplate reader. This assay was applied to quantify Vimentin secretion.

### Cell Counting Kit-8 (CCK-8)

HBMECs were cocultured with LUAD cell lines (A549, NCI-H1975, and PC-9) and then seeded at a density of 2,000 cells per well in 96-well plates. At predetermined time points (0, 24, 48, and 72 h), CCK-8 reagent (10 µL) was added to each well, and the samples were incubated at 37 °C for 2 h. The optical density of each well was measured at 450 nm using a Varioskan LUX microplate reader to evaluate cell viability and proliferation.

### 5-Ethynyl-2'-Deoxyuridine Assay (EdU Assay)

HBMECs were cocultured with LUAD cell lines and then seeded at a density of 2×10^4^ cells/mL in 6-well plates. EdU reagent (20 µM) was added to the culture medium and incubated at 37 °C for 2 hours to allow incorporation into newly synthesized DNA. Cells were fixed with 4% paraformaldehyde for 15 min, permeabilized using Triton X-100, and stained with click additive solution for 30 min in the dark to facilitate the visualization of EdU-positive cells. Following nuclear counterstaining with DAPI, the proportion of EdU-positive cells relative to the total number of DAPI-positive cells was quantified utilizing a Nikon Ti2 fluorescence microscope.

### Western Blotting

Total cellular proteins were extracted using radioimmunoprecipitation assay lysis buffer supplemented with protease inhibitors to prevent degradation. Protein lysates were resolved via sodium dodecyl sulfate‒polyacrylamide gel electrophoresis and transferred to polyvinylidene fluoride membranes. The membranes were blocked with 5% skim milk for 1 h to prevent nonspecific binding, followed by incubation with specific primary antibodies at 4 °C overnight. The primary antibodies used included those against S100A9 (PA5-79949, Thermo), ZO-1 (61-7300, Thermo), Occludin (ab216327, Abcam), Claudin-5 (34-1600, Thermo), ITGB1 (ab52971, Abcam), FAK (ab40794, Abcam), Vimentin (ab137321, Abcam), VE-cadherin (2500, CST), N-cadherin (66219-1-Ig, Proteintech), α-SMA (ab124964, Abcam), P62 (ab109012, Abcam), p-Beclin1 (AF7386, Affinity), Beclin1 (ab302669, Abcam), LC3 (ab192890, Abcam), Flag (ab205606, Abcam), V5 (14440-1-AP, Proteintech), Ub-K48 (ab140601, Abcam), Ub-WT (ab134953, Abcam), Flag (F1804, Sigma), and USP33 (20445-1-AP, Proteintech). Following thorough washing with Tris-buffered saline containing Tween-20, the membranes were incubated with HRP-conjugated secondary antibodies. The protein bands were finally visualized with an enhanced chemiluminescence reagent (MA0186-2; Meilunbio).

### Wound Healing Assay

HBMECs were cocultured with LUAD cell lines and grown to 90% confluence in 6-well plates to establish a uniform monolayer. A linear scratch wound was generated on the cell monolayer with a sterile pipette tip. The cells were subsequently maintained in media supplemented with 10% FBS to mitigate proliferation-induced artifacts. Cellular migration into the wound zone was quantified at 0, 24, and 48 hours with an XD-202 inverted microscope to evaluate migratory capacity.

### Transwell Migration Assay

A549, NCI-H1975, and PC-9 cells (2×10⁵ cells/well) were inoculated into the upper compartments of Transwell inserts containing serum-free medium, and medium supplemented with 10% FBS was added to the lower compartment as a chemoattractant. After 24 hours of incubation, nonmigratory cells on the apical membrane surface were carefully removed using a cotton swab. Migratory cells on the basolateral surface were fixed with 4% paraformaldehyde, dyed with 0.1% crystal violet, and quantified with an XD-202 inverted microscope.

### Transwell Invasion Assay

To assess invasive potential, cells (2 × 10⁵/well) were inoculated into the upper compartments of a Transwell chamber that had been precoated with Matrigel and incubated at 37 °C for 5 hours to facilitate extracellular matrix traversal. The upper compartment contained serum-free medium, whereas the lower compartment contained medium supplemented with FBS. Following a 24-hour incubation, the cells that had penetrated the Matrigel matrix were immobilized, stained, and enumerated according to the protocol established for the migration assay.

### Colony Formation Assay

A549, NCI-H1975, or PC-9 cells were inoculated into gelatin-coated 6-well plates and maintained for 14 days under standard culture conditions (37 °C, 5% CO₂) to facilitate colony formation. After incubation, the cells were gently rinsed with PBS, fixed for 15 minutes, stained with crystal violet for 15 minutes, and quantified using an XD-202 microscope and ImageJ software to assess clonogenic survival.

### Terminal Deoxynucleotidyl Transferase dUTP Nick End Labeling (TUNEL Staining)

Stable cell lines (A549, NCI-H1975, and PC-9) were subjected to various experimental treatments and then fixed with 4% (w/v) formaldehyde. DNA fragmentation, which is characteristic of apoptosis, was detected via TUNEL staining using a commercial kit (C1090; Beyotime) with 1 hour of reaction at 37 °C. The cell nuclei were counterstained with DAPI (P0131; Beyotime) for 5 minutes to visualize the total cellular population. The samples were subsequently visualized microscopically using a Nikon Ti2 fluorescence microscope.

### Tube Formation Assay

HBMECs were cocultured with LUAD cell lines (A549, H1975, and PC-9) at the optimized densities in 6-well plates, and the culture medium was replaced every 48 hours. For the assay, Matrigel (200 µL/well) was applied to 24-well plates and allowed to polymerize at 37 °C for 1 hour. Cells (1×10⁵/well) were subsequently seeded onto the substrate, gently agitated to ensure uniform distribution, and cultured. Capillary-like vascular network formation was visualized, and the morphological parameters, including branch number and total tube length, were quantitatively assessed.

### Immunofluorescence

Cells cultured in 12-well plates were fixed with 4% paraformaldehyde for 10 minutes at ambient temperature, followed by permeabilization using 0.5% Triton X-100 for 10 minutes. Nonspecific binding was subsequently attenuated by blocking with goat serum for 2 hours. Primary antibodies against tight junction proteins (ZO-1, Claudin-5, and Occludin) were added, and the samples were incubated overnight at 4 °C. After rinsing, an AF594-conjugated secondary antibody was added, and the cells were incubated for 2 hours in the dark. The nuclei were labeled with DAPI for 10 minutes, after which the cells were mounted with anti-fade medium. The fluorescence was detected after the samples were sealed with clear nail polish.

### Lysosome Detection

LysoTracker Red was diluted to a final concentration of 60 nM in prewarmed culture medium and added to cells for 60 minutes of incubation at 37 °C to specifically label acidic organelles. After the incubation, the medium was replaced with fresh medium, and the subcellular distribution of the lysosomes was visualized and documented via confocal microscopy.

### Detection of Sodium Fluorescein (F-Na) Permeability

HBMECs were inoculated into Transwell inserts at a density of 1×10⁵ cells/mL and cocultured with LUAD cells. Upon reaching approximately 70% confluence, 0.2 mL of a 1 mg/mL solution of sodium fluorescein was added to the apical chamber for 2 hours of incubation. The fluorescence intensity of the medium in the basolateral chamber was quantified with a microplate reader. The F-Na⁺ permeability was extrapolated from a calibration curve established across the concentration gradient of 4 to 1000 µg/mL.

### ROS Detection

HBMECs cocultured with LUAD cells were incubated with 10 µM 2',7'-dichlorofluorescein diacetate (DCFH-DA) in serum-deprived medium. For *in situ* loading, the cells were exposed to the fluorescent probe at 37 °C for 20 minutes, followed by rinsing to eliminate residual dye. An identical protocol was employed for postcollection loading. ROS levels were quantified via confocal microscopy using excitation and emission wavelengths of 488 nm and 525 nm, respectively, revealing a pronounced increase in fluorescence in cells stimulated for 30 minutes.

### 5,5′,6,6′-Tetrachloro-1,1′,3,3′-Tetraethylbenzimidazolylcarbocyanine Iodide (JC-1) Staining

HBMECs cocultured with LUAD cells were rinsed with PBS and incubated with 1 mL of JC-1 staining solution (Beyotime C2003) at 37 °C for 20 minutes to evaluate the mitochondrial membrane potential. The cells were washed twice with buffer, resuspended in 2 mL of medium, and subsequently visualized with a Leica DM3000 LED confocal microscope to discriminate between healthy (red fluorescence) and depolarized (green fluorescence) mitochondria.

### Transcriptome Sequencing Analysis

The raw sequencing data were subjected to quality control with fastp software to remove adapter sequences, poly-N-containing regions, and low-quality sequencing reads. Key quality control metrics, specifically Q20, Q30, and the GC content, were determined to verify data integrity. Clean reads were mapped to the reference genome utilizing HISAT2. Transcriptional abundance, quantified as fragments per kilobase of transcript sequence per million mapped reads (FPKM), was determined with featureCounts. Differential expression analysis was performed with DESeq2 for replicate samples and edgeR for nonreplicate samples with statistical significance thresholds of an adjusted P value (adj. P) ≤ 0.05 and an absolute log₂ fold change (|log₂FC|) ≥ 1. The functional enrichment of differentially expressed genes was evaluated by Gene Ontology (GO) and the Kyoto Encyclopedia of Genes and Genomes (KEGG) analyses with the clusterProfiler package. Subsequent pathway investigation was performed utilizing gene set enrichment analysis (GSEA). Protein‒protein interaction (PPI) networks were constructed utilizing the STRING and Diamond databases. Furthermore, alternative splicing events were investigated with rMATS, whereas SNP identification and annotation were performed using GATK and SnpEff.

### Immunoprecipitation-Mass Spectrometry (IP‒MS)

Mass spectrometric data were processed and peak lists were generated utilizing the Data Analysis 4.0 platform. Database searches were performed with the MASCOT 2.2.06 algorithm against the curated Swiss-Prot knowledgebase. The search parameters included trypsin digestion with one missed cleavage allowed, fixed cysteine carbamidomethylation, variable methionine oxidation, restriction to *Homo sapiens*, a peptide mass tolerance of 10 ppm, and a fragment ion tolerance of 0.6 Da. Protein identification was based on statistically significant MOWSE scores. Nonspecific cleavage products were identified utilizing a semispecific search algorithm. Specific interacting proteins were carefully identified by excluding those also detected in the IgG control cohorts.

### Protein Half-Life Assay

293T cells were transfected with V5-USP33-OE or vector control constructs and subsequently exposed to MG132 (10 µM), cycloheximide (CHX, 100 µM), or DMSO (1:5000) for 0, 4, 8, or 12 hours. The samples were then treated with CHX (100 µM) for 0, 4, 8, or 12 hours to evaluate the protein half-life, along with MG132 and DMSO as controls. Cells were harvested at designated time points for Western blotting to determine the degradation kinetics and half-life of S100A9.

### Co-IP

Protein A/G beads were subjected to preclearance with control IgG in lysis buffer at 4 °C for 30 minutes to minimize nonspecific interactions. The beads were subsequently sequentially washed with high-salt and low-salt RIPA buffers. Cell lysates (500 μg) were reserved as input controls. The supernatants were incubated with the washed beads at 4 °C for 30 minutes. Following six thorough washes, the beads were resuspended in 2× loading buffer, denatured by boiling for 10 minutes, and cryopreserved at -20 °C. To facilitate targeted immunoprecipitation, the supernatant was incubated with 25 μg of a specific antibody (anti-Flag/V5/S100A9) overnight at 4 °C. Protein A/G beads were added and incubated with the sample for 3 hours with rotation to facilitate immune complex capture. After being washed, the samples were processed as described previously. The protein concentration was quantified via a BCA assay, and target proteins were resolved via Western blotting.

### Tumor Formation in Nude Mice

Thirty-two female BALB/c nude mice, aged 6-8 weeks and reared under specific pathogen-free (SPF) conditions, were maintained in individually ventilated cages equipped with sterile bedding within a rigorously controlled environment (20-26 °C, 40-70% humidity, 12-hour light/dark cycle). Standard laboratory chow and water were supplied ad libitum. A549 and H1975 cells that had been stably transfected with the empty vector or S100A9-OE construct were cultured in DMEM supplemented with 10% FBS and subjected to puromycin selection. Tumorigenesis was initiated via subcutaneous administration of 5×10^6^ cells into the flank of each mouse (n = 8 per group).

### Small Animal Imaging Experiments

Twenty-four male NOD-SCID mice, aged 6-8 weeks and classified as specific pathogen-free, were housed under rigorously controlled environmental conditions. The stable A549 and H1975 cell lines were cultured in accordance with the previously described protocol. To establish a metastasis model, 24 anesthetized mice received a left cardiac ventricle injection of 1×10⁵ cells suspended in 20 µL (6 mice per group). Following a three-week interval to facilitate metastatic colonization, 100 µL of substrate was administered via the tail vein, and bioluminescence imaging was performed to detect and quantify the metastatic lesions.

### Evans Blue Staining

To assess blood‒brain barrier permeability, a solution comprising 1% Evans blue (20 mL/kg, Solaibio) and heparinized saline (20 U/mL) was formulated. Anesthetized subjects received an intravenous injection of the dye solution (2 mL/kg) via the tail vein. Subsequently, the thoracic cavity was exposed to facilitate transcardial perfusion, physiological saline (0.9% sodium chloride) was gently infused through the left ventricular apex, and an incision was made into the right atrium to ensure adequate drainage. Perfusion was sustained until the effluent became clear, indicating that the intravascular dye had been removed. Subsequently, the brain tissues were harvested and imaged via stereomicroscopy to visualize the staining pattern.

### Statistical Analysis

All experiments were conducted in triplicate. Statistical analyses were performed using GraphPad 8.0 software. Data from multiple groups were compared via one-way analysis of variance (ANOVA) with Tukey's post hoc test, whereas data from two groups were compared using Student's t test. A p value < 0.05 was considered to indicate statistical significance. The data are presented as the mean ± standard deviation (SD). ns, not significant; *P < 0.05; **P < 0.01; ***P < 0.001.

## Results

### Significance of S100A9 Expression in LUAD Prognosis and Validation of Stable S100A9 Expression in LUAD Cell Lines

**Figure 1:**
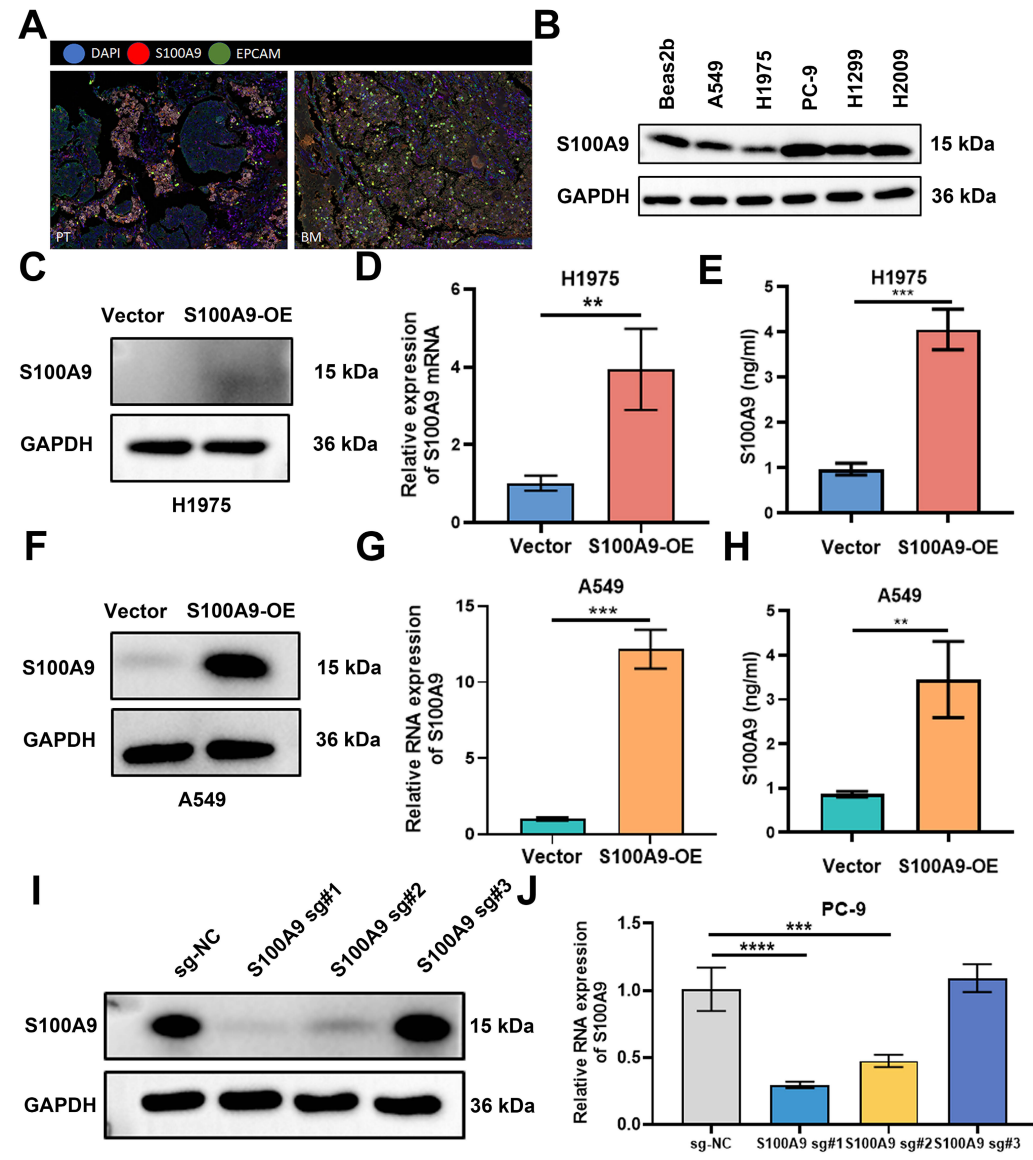
Expression of S100A9 in LUAD samples and its association with prognosis and validation of the stably transfected S100A9 LUAD cell lines. **(A)** Multiplex immunohistochemistry showing S100A9-positive and EPCAM-positive cells in brain metastases of LUAD patients. Blue: DAPI; red: S100A9; green: EPCAM. **(B)** Western blot showing S100A9 protein levels in human bronchial epithelial (BEAS-2B) cells and NSCLC cells (A549, H1975, PC-9, H1299, and H2009). **(C, F, I)** Western blot showing S100A9 protein levels in H1975 cells (C) from the Vector and S100A9-OE groups, and in A549 cells (F) and PC-9 cells (I) from the sg-NC and sg-S100A9 groups. n = 3 independent biological replicates. Data are presented as mean ± SD. **(D, E, G, H, J)** RT‒qPCR and ELISA results showing S100A9 RNA levels (D, G) and secreted S100A9 protein levels (E, H) in H1975 cells (D, E) and A549 cells (G, H) from the Vector and S100A9-OE groups, and S100A9 RNA levels in PC-9 cells (J) from the sg-NC and sg-S100A9 groups. **P < 0.01; ***P < 0.001; ****P < 0.0001; n = 3 independent biological replicates. Data are presented as mean ± SD. Data were analyzed by one-way ANOVA followed by Tukey's post hoc test. Full-length blots/gels are presented in the raw image.

Multiplex immunohistochemistry revealed pronounced enrichment of EPCAM-positive epithelial cells in LUAD brain metastasis specimens, and S100A9 exhibited frequent colocalization with EPCAM in these metastases **(Fig. 1A)**. To investigate the potential role of S100A9 in LUAD pathogenesis, its expression was quantified across a panel of cell lines, including human bronchial epithelial cells (BEAS-2B) and several LUAD cell lines (A549, H1975, PC-9, H1299, and H2009). S100A9 expression was significantly reduced in A549 and H1975 cells and was highest in PC-9 cells **(Fig. 1B)**.

To elucidate the mechanistic contribution of S100A9 to LUAD brain metastasis, gain-of-function and loss-of-function cellular models were established. A549 and H1975 cells were transduced with either a control vector or an S100A9-OE lentiviral construct. Subsequent RT‒qPCR and immunoblotting analyses revealed substantial increases in S100A9 transcription and translation in the S100A9-OE group compared with the Vector group. Moreover, the amount of secreted S100A9 protein was significantly greater in the S100A9-OE groups than in the Vector groups **(Fig. 1C-H)**. Furthermore, an sgRNA-mediated CRISPR-Cas9 system was used to knock out S100A9 in PC-9 cells. The knockout efficiency was rigorously confirmed by RT‒qPCR and Western blotting in the sg-NC, S100A9 sg#1, S100A9 sg#2, and S100A9 sg#3 groups, and the highest knockout efficiency was observed with S100A9 sg#1** (Fig. 1I, J)**.

### Effects of S100A9 Overexpression on the Tumorigenicity and Brain Metastasis of H1975 and A549 Cells

A panel of *in vitro* functional assays, including CCK-8 proliferation, colony formation, EdU incorporation, Transwell migration and invasion, wound healing, and TUNEL apoptosis assays, were performed with A549 and H1975 cells. Comparative analysis revealed that, compared with the Vector group, the proliferative, migratory, and invasive capacities of cells in the S100A9-OE group were significantly increased, whereas apoptosis was attenuated (Supplementary Fig. 1 and 2). Conversely, PC-9 cells in the S100A9 sg#1 group exhibited markedly attenuated proliferation, migration, and invasion but increased apoptosis compared with the sg-NC group (Supplementary Fig. 3).

**Figure 2:**
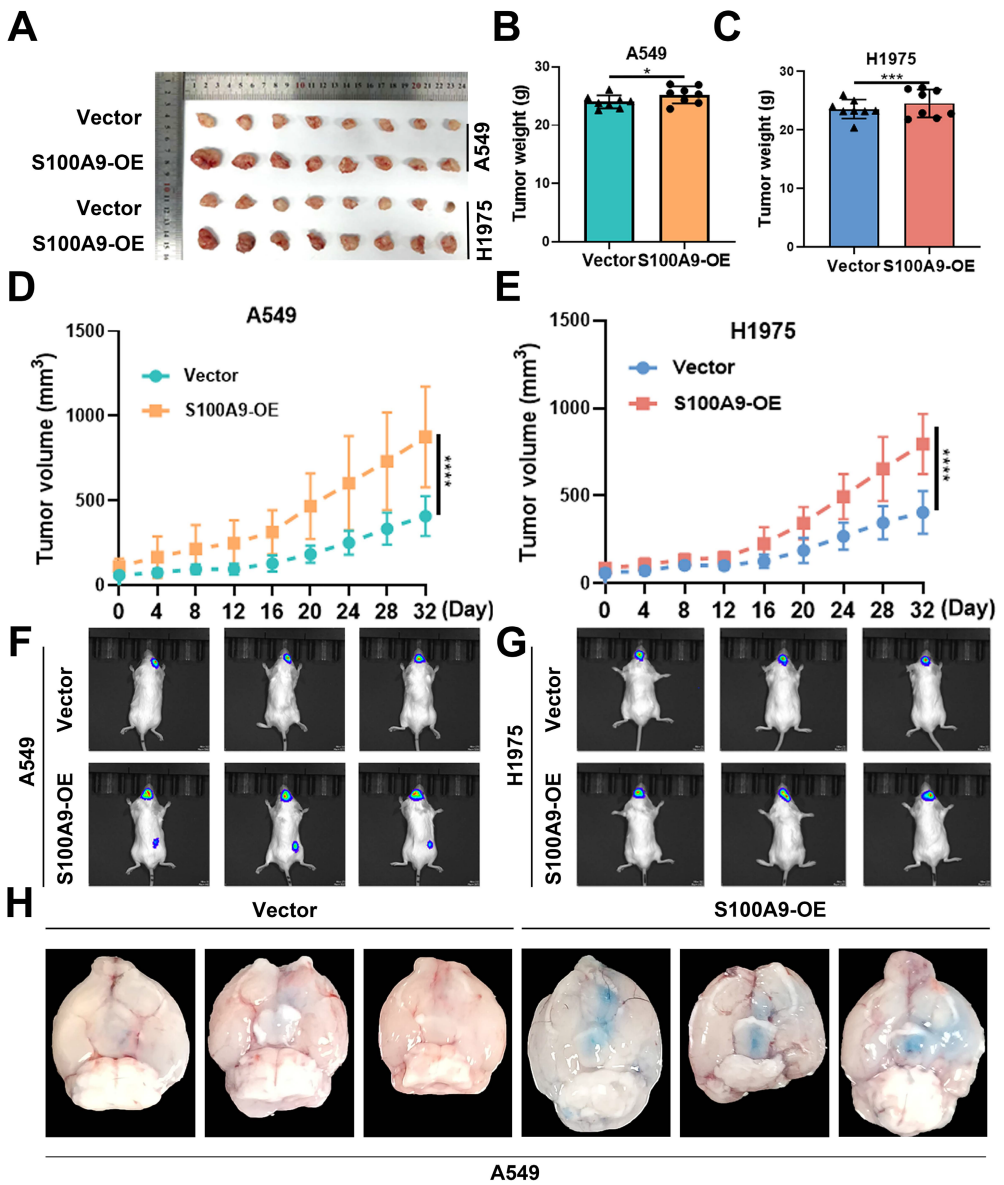
Effects of S100A9-OE on tumorigenesis *in vivo* and on the brain metastatic potential of H1975 and A549 cells. **(A-H)** A549 cells and H1975 cells were divided into a Vector group and an S100A9-OE group and used in the following experiments. **(A)** A549 and H1975 cells were injected into BALB/c nude mice to establish xenograft models. On day 32, the mice were euthanized, and the tumors were dissected and photographed (n = 8 per group). **(B, C)** Weights of tumors formed 32 days after subcutaneous injection of 5×10⁶ A549 cells (B) and H1975 cells (C) into the right axilla. *P < 0.05; ***P < 0.001; n = 8 independent biological replicates. Data are presented as mean ± SD. **(D, E)** Tumor volumes measured with a Vernier caliper at the indicated time points after subcutaneous injection of 5×10⁶ A549 cells (D) and H1975 cells (E) into the right axilla. ****P < 0.0001; n = 8 independent biological replicates. Data are presented as mean ± SD. **(F, G)** A549 cells (F) and H1975 cells (G) stably transfected with Vector-Luc or S100A9-Luc (expressing luciferase) (n = 6 per group) were injected into NOD-SCID mice via left cardiac ventricle injection, followed by small animal *in vivo* imaging. n = 6 independent biological replicates. Data are presented as mean ± SD. **(H)** The brain region with the strongest luminescence signal was imaged, and blood-brain barrier permeability was evaluated by Evans blue extravasation (n = 4 per group). n = 4 independent biological replicates. Data are presented as mean ± SD. Statistical significance was determined by Student's t test and two-way ANOVA followed by Bonferroni post hoc correction.

To rigorously assess tumorigenic potential *in vivo*, we subcutaneously inoculated 5×10⁶ lentivirus-treated A549 or H1975 cells from the S100A9-OE and Vector groups into the right axillary region of BALB/c nude mice. Tumors were surgically excised and imaged on day 32 post-inoculation **(Fig. 2A)**. Compared with the Vector group, both tumor weight and tumor volume were significantly increased in the S100A9-OE group **(Fig. 2B-E)**.

To evaluate brain metastatic potential, A549 and H1975 cells stably expressing S100A9-Luc or Vector-Luc were inoculated into the left ventricle. *In vivo* bioluminescence imaging demonstrated that intracranial photon flux was significantly greater in the S100A9-OE group than in the Vector group **(Fig. 2F, G)**, confirming that S100A9 functions as a critical promoter of both tumor growth and brain metastasis in this LUAD model.

To assess BBB integrity, an Evans blue extravasation assay was performed in nude mice. Quantitative analysis revealed a marked increase in Evans blue extravasation in the S100A9-OE group compared with the Vector group, suggesting that S100A9 overexpression substantially increases BBB permeability and facilitates the extravasation of Evans blue into the brain parenchyma **(Fig. 2H)**.

### S100A9 Modulates HBMEC Function in the BBB to Facilitate LUAD Metastasis

**Figure 3:**
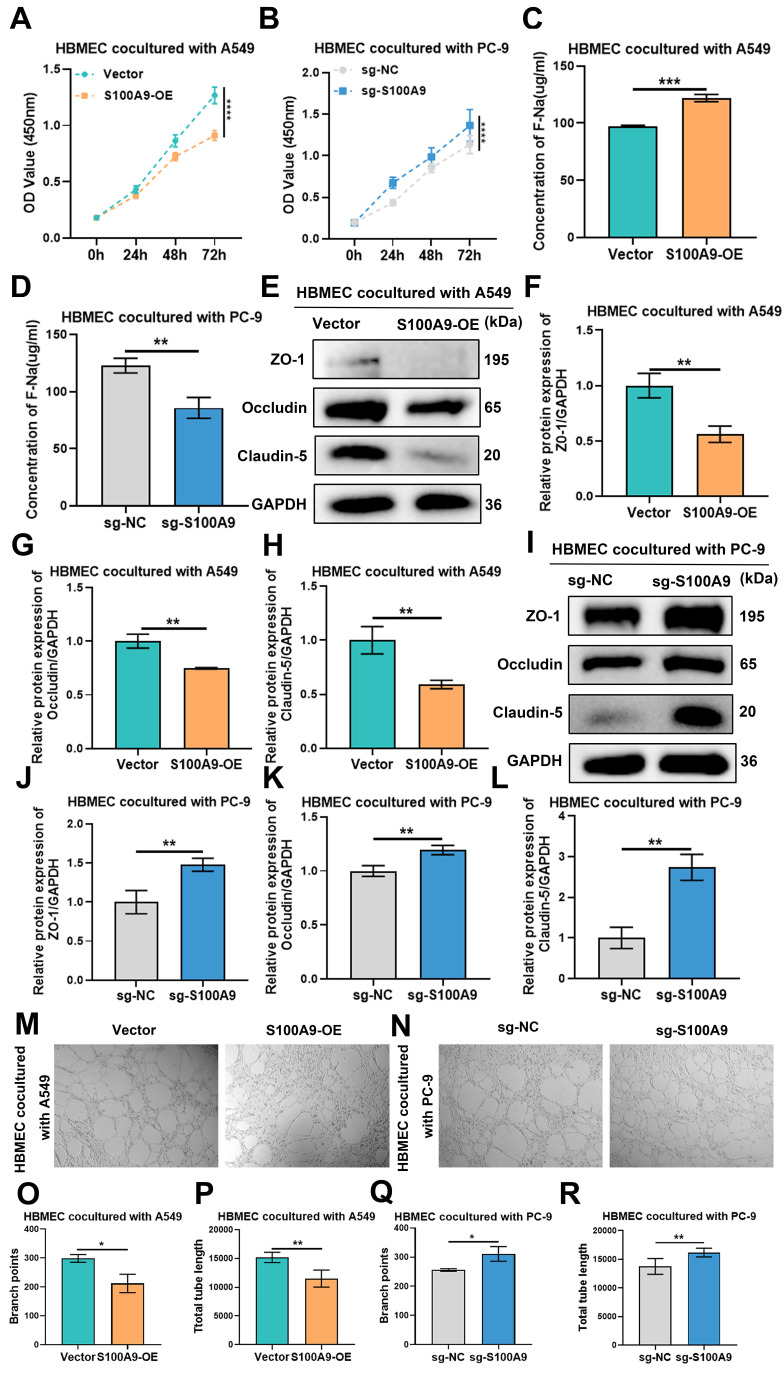
S100A9 mediates BBB dysfunction by regulating the malignant behavior of LUAD cells. **A549** cells from the Vector group and S100A9-OE group and **PC-9** cells from the sg-NC group and sg-S100A9 group were used in the following experiments. HBMECs and LUAD cells were cocultured in a Transwell system (0.4 μm) for 48 hours. **(A, B)** CCK-8 assay results showing OD values at 450 nm of HBMECs after 0, 24, 48, and 72 h of co-culture with A549 (A) or PC-9 (B) cells. ****P < 0.0001; n = 3 independent biological replicates. Data are presented as mean ± SD. **(C, D)** Na-F permeability assay results showing the permeability of HBMECs co-cultured with A549 (C) or PC-9 (D) cells. **P < 0.01; ***P < 0.001; n = 3 independent biological replicates. Data are presented as mean ± SD. **(E-L)** Western blot showing the expression levels of the tight junction proteins ZO-1, Occludin, and Claudin-5 in HBMECs co-cultured with A549 (E-H) or PC-9 cells (I-L). **P < 0.01; n = 3 independent biological replicates. Data are presented as mean ± SD. **(M-R)** Tube formation assay results showing the number of branches and total tube lengths formed by HBMECs co-cultured with A549 (M, O, P) or PC-9 cells (N, Q, R). *P < 0.05; **P < 0.01; n = 3 independent biological replicates. Data are presented as mean ± SD. Data were analyzed by Student's t test and two-way ANOVA followed by Bonferroni post hoc correction. Full-length blots/gels are presented in the raw image.

Leveraging an established coculture paradigm, we investigated the paracrine influence of LUAD cells with different levels of S100A9 expression on HBMECs. CCK-8 assays revealed that the proliferative capacity of HBMECs cocultured with A549 cells in the **S100A9-OE group** decreased in a time-dependent manner compared with that in the **Vector group (Fig. 3A)**, and a similar trend was observed in the **S100A9-OE group** of H1975 cells **(Sup Fig. 4A)**. Compared with the **sg-NC group**, the group of HBMECs cocultured with PC-9 cells from the **sg-S100A9 group** exhibited significantly increased proliferative activity **(Fig. 3B)**, suggesting that S100A9 potentiates the LUAD-induced inhibition of endothelial growth.

F-Na+ permeability assays revealed a marked increase in HBMEC permeability upon coculture with A549 cells in the **S100A9-OE group** compared with those in the **Vector group (Fig. 3C)**, and HBMECs cocultured with H1975 cells from the **S100A9-OE group** exhibited similar alterations **(Sup Fig. 4B)**. Conversely, in contrast to the **sg-NC group**, HBMECs cocultured with PC-9 cells from the **sg-S100A9 group** exhibited a marked decrease in permeability **(Fig. 3D)**, suggesting that S100A9 facilitates endothelial barrier dysfunction.

Western blot analysis of total protein extracted from HBMECs cocultured with A549 cells revealed that ZO-1, Occludin, and Claudin-5 expression was significantly lower in HBMECs from the **S100A9-OE group** than in those from the **Vector group (Fig. 3E-H)**, and HBMECs cocultured with H1975 cells in the **S100A9-OE group** exhibited similar alterations **(Sup Fig. 4C-F)**. Compared with those in the **sg-NC group**, the protein expression levels of the tight junction proteins ZO-1, Occludin, and Claudin-5 were significantly elevated in the groups of HBMECs cocultured with PC-9 cells in the **sg-S100A9 group (Fig. 3I-L)**, underscoring the pivotal role of S100A9 in the disruption of tight junctions.

Furthermore, the results of the tube formation assays revealed that compared with HBMECs cocultured with A549 cells from the **Vector group**, HBMECs cocultured with A549 cells from the **S100A9-OE group** presented fewer branching points and shorter total tube lengths **(Fig. 3M, O, P)**, and similar results were observed for the HBMECs cocultured with H1975 cells from the **S100A9-OE group (Sup Fig. 4G-I)**. Compared with those in the **sg-NC group**, the number of branch points and total tube lengths of HBMECs cocultured with PC-9 cells from the **sg-S100A9 group** were significantly greater **(Fig. 3N, Q, R)**, demonstrating that S100A9 impairs the angiogenic capacity of HBMECs.

### S100A9 Overexpression Modulates Transcriptomic Alterations and Signaling Pathways in A549 Cells

**Figure 4:**
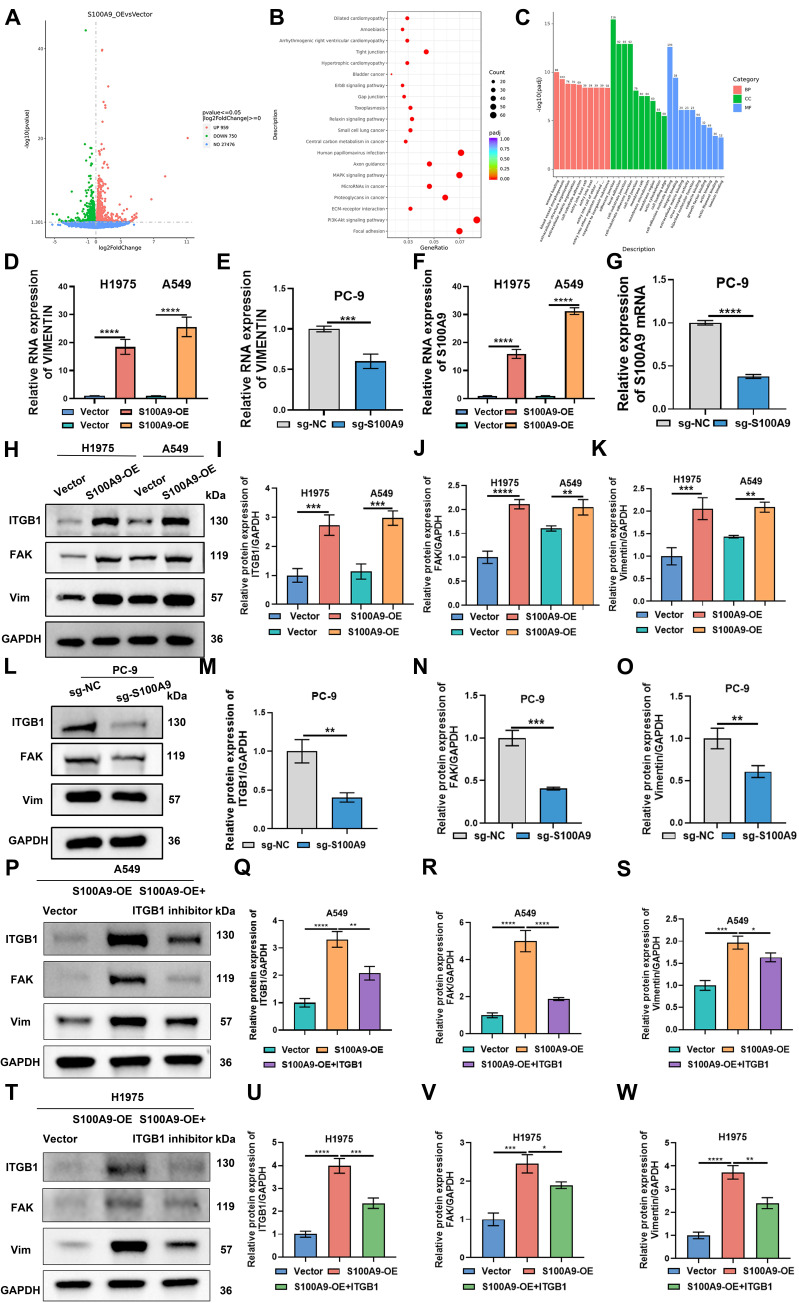
Effects of S100A9 overexpression on the transcriptome, differentially expressed genes, and signaling pathways in A549 cells. **(A)** Volcano plot showing genes differentially expressed between the Vector and S100A9-Luc groups of A549 cells. Each data point represents a single gene from three independent biological replicates. **(B)** KEGG analysis of enriched signaling pathways between the S100A9-Luc and Vector groups. Notably, the focal adhesion and ECM signaling pathways were regulated by S100A9. **(C)** GO analysis of differences in biological processes, cellular components, and molecular functions between the Vector and S100A9-Luc groups.** (D-G)** RT‒qPCR results showing RNA levels of S100A9 and Vimentin in A549, H1975, and PC-9 cells. ***P < 0.001; ****P < 0.0001; n = 3 independent biological replicates. Data are presented as mean ± SD. **(H-O)** Western blot showing protein expression levels of ITGB1, FAK, and Vimentin in A549 or H1975 (H-K) and PC-9 (L-O) cells. **P < 0.01; ***P < 0.001; ****P < 0.0001; n = 3 independent biological replicates. Data are presented as mean ± SD. **(P-W)** Western blot showing protein expression levels of ITGB1, FAK, and Vimentin in A549 or H1975 cells between Vector group, S100A9-OE group and S100A9-OE+ITGB1 inhibitor group. **P < 0.01; ***P < 0.001; ****P < 0.0001; n = 3 independent biological replicates. Data are presented as mean ± SD. Statistical significance was determined by Student's t test and one-way ANOVA followed by Tukey's post hoc test. Full-length blots/gels are presented in the raw image.

To elucidate the transcriptional reprogramming mediated by S100A9 overexpression in A549 cells, we performed high-throughput transcriptome sequencing. Compared with the **Vector group**, a total of 750 downregulated and 959 upregulated genes were identified in the **S100A9-OE group**; among these genes, Vimentin was significantly upregulated **(Fig. 4A)**. KEGG pathway enrichment analysis revealed increased activity in focal adhesion and extracellular matrix (ECM) signaling pathways in the **S100A9-OE group** compared with the **Vector group**, and Vimentin was implicated in these critical biological processes **(Fig. 4B)**. GO analysis revealed that cell adhesion-related components were significantly enriched in A549 cells in the **S100A9-OE group** compared with those in the **Vector group (Fig. 4C)**.

To empirically validate the S100A9-mediated regulation of Vimentin in LUAD models, RT‒qPCR was performed, and the results revealed that for both A549 and H1975 cells, compared with those in the **Vector group**, the RNA levels of S100A9 and Vimentin in the **S100A9-OE group** were significantly greater. Conversely, compared with those in the **sg-NC group**, the RNA levels of S100A9 and Vimentin in the **sg-S100A9 group** were significantly lower **(Fig. 4D-G)**. Western blot analysis revealed that compared with those in the **Vector group**, the protein expression levels of ITGB1, Focal Adhesion Kinase, and Vimentin in the **S100A9-OE group** were greater. In PC-9 cells, the protein expression of ITGB1, Focal Adhesion Kinase, and Vimentin proteins was significantly lower in the **sg-S100A9 group** than in the** sg-NC group (Fig. 4H-O)**. Collectively, these results underscore the pivotal role of S100A9 in modulating the cell adhesion machinery, particularly through the regulation of Vimentin expression, during LUAD pathogenesis.

To investigate the role of the ITGB1 pathway, we treated A549 and H1975 cells with an ITGB1 inhibitor. Western blot analysis demonstrated a significant reduction in the expression levels of ITGB1, FAK, and Vimentin proteins in the **S100A9-OE+ITGB1 inhibitor group** compared to the **S100A9-OE group** across both cell lines **(Figure 4P-W)**. Cell viability was evaluated using the CCK-8 assay, revealing that the **S100A9-OE+ITGB1 inhibitor group** exhibited markedly diminished viability relative to the **S100A9-OE group** in both A549 and H1975 cells **(Sup Figure 4J-K)**. Transwell assays were conducted to assess migration and invasion capabilities. Results indicated a substantial decrease in the number of migrating and invading cells within the **S100A9-OE+ITGB1 inhibitor group** when compared to the **S100A9-OE group** for both cell types **(Sup Figure 4L-Q)**. Collectively, these findings underscore the pivotal role of the ITGB1 pathway in modulating S100A9-induced effects on cellular proliferation, migration, and invasion in A549 and H1975 cells.

### S100A9 Exacerbates BBB Dysfunction and Promotes Endothelial-Mesenchymal Transition (EndMT) in HBMECs by Upregulating Vimentin Expression

**Figure 5:**
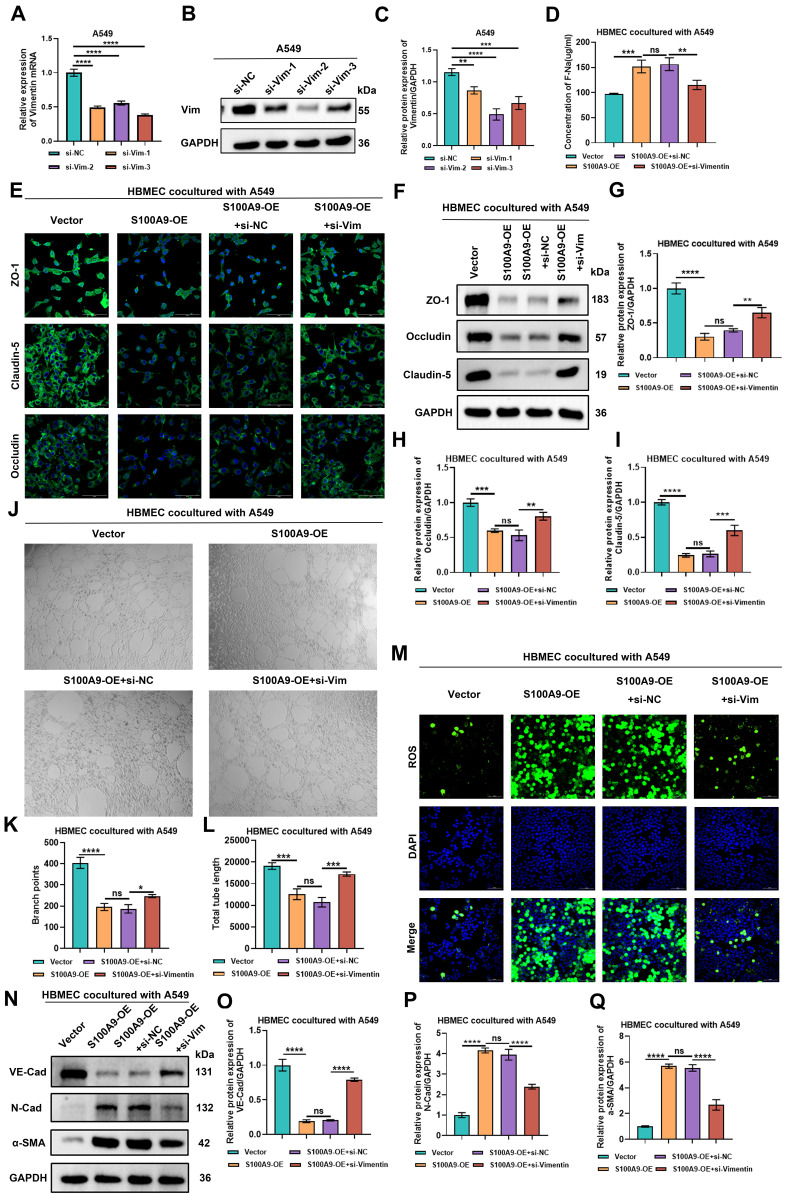
S100A9 exacerbates BBB damage and promotes End-MT in HBMECs by upregulating Vimentin expression. **Groups (A-Q):** The following groups of cells were used in the experiments: Vector group A549 cells cocultured with HBMECs; S100A9-OE group A549 cells cocultured with HBMECs; S100A9-OE+si-NC group A549 cells cocultured with HBMECs; and S100A9-OE+si-Vimentin group A549 cells cocultured with HBMECs. **(A-C)** RT‒qPCR (A) and Western blotting (B, C) results showing Vimentin RNA and protein levels in A549 cells transfected with si-1, si-2, and si-3. **P < 0.01; ***P < 0.001; ****P < 0.0001; ns, not significant; n = 3 independent biological replicates. Data are presented as mean ± SD. **(D)** Na-F permeability assay results showing the permeability of HBMECs co-cultured with A549 cells. **P < 0.01; ***P < 0.001; ns; n = 3 independent biological replicates. Data are presented as mean ± SD. **(E)** Immunofluorescence staining analysis of ZO-1, Claudin-5, and Occludin fluorescence intensities in HBMECs co-cultured with A549 cells. **(F-I)** Western blot showing protein expression levels of ZO-1, Claudin-5, and Occludin in HBMECs co-cultured with A549 cells. **P < 0.01; ***P < 0.001; ****P < 0.0001; ns; n = 3 independent biological replicates. Data are presented as mean ± SD. **(J-L)** Tube formation experiments showing the number of branch points and tube lengths of HBMECs cocultured with A549 cells. *P < 0.05; ***P<0.001; ****P < 0.0001; N=3 independent biological replicates; ns, not significant; Data are the mean ± SD. **(M)** ROS levels in HBMECs cocultured with A549 cells. **(N-Q)** Western blot showing protein expression levels of VE-Cadherin, N-Cadherin, and α-SMA in HBMECs co-cultured with A549 cells. ****P < 0.0001; ns; n = 3 independent biological replicates. Data are presented as mean ± SD. Statistical significance was determined by one-way ANOVA followed by Tukey's post hoc test. Full-length blots/gels are presented in the raw image.

To investigate the mechanistic relationship between S100A9 and Vimentin in HBMECs, we performed siRNA-mediated knockdown of Vimentin expression. RT‒qPCR and immunoblotting confirmed that in the **siVIM-1, siVIM-2, and siVIM-3 groups** of A549 cells, the silencing efficiency of Vimentin was significantly reduced compared with that of the **si-NC group**, with siVIM-2 producing the greatest silencing efficiency **(Fig. 5A-C)**; similar results were obtained with H1975 cells **(Sup Fig. 5A-C)**.

Sodium fluoride (F-Na+) permeability assays revealed that HBMECs cocultured with A549 cells in the** S100A9-OE group** exhibited significantly increased ion flux compared with coculture with cells in the **Vector group**. No significant difference in sodium ion flux was observed between the **S100A9-OE group** and the **S100A9-OE+si-NC group**. However, compared with the **S100A9-OE+si-NC group**, the **S100A9-OE+si-Vimentin group** showed a significant reduction in sodium ion flux **(Fig. 5D)**. Similar findings were observed with the H1975 coculture systems **(Sup Fig. 5D)**, suggesting the presence of a Vimentin-dependent regulatory mechanism.

Immunofluorescence and Western blot analyses revealed that the fluorescence intensity and protein expression levels of the tight junction proteins ZO-1, Claudin-5, and Occludin were significantly lower in HBMECs cocultured with A549 cells from the **S100A9-OE group** upon coculture with cells from the **Vector group**. No significant differences in fluorescence intensity or ZO-1, Claudin-5, or Occludin protein expression were detected between the **S100A9-OE group** and the **S100A9-OE+si-NC group**. However, compared with those in the **S100A9-OE+si-NC group**, the fluorescence intensity and protein expression of ZO-1, Claudin-5, and Occludin were significantly greater in the **S100A9-OE+si-Vimentin group (Fig. 5E-I)**, and a similar trend was detected with the H1975 cell model systems** (Sup Fig. 5E-I),** indicating that S100A9 compromises BBB barrier integrity by modulating Vimentin expression.

The tube formation assays demonstrated that HBMECs cocultured with A549 cells from the **S100A9-OE group** exhibited significantly fewer branch points and total tube lengths compared with HBMECs cocultured with A549 cells from the **Vector group**. Compared with those in the **S100A9-OE group**, the cells in the **S100A9-OE+si-NC group** did not significantly differ in terms of these metrics. Conversely, compared with those in the **S100A9-OE+si-NC** group, the number of branch points and total tube lengths in the **S100A9-OE+si-Vimentin group** were restored **(Fig. 5J-L)**. The results were similar upon coculture with H1975 cells **(Sup Fig. 5J-L)**. These data suggest that S100A9 inhibits the angiogenic capacity of HBMECs via Vimentin signaling.

Immunofluorescence detection of ROS levels revealed that HBMECs cocultured with A549 cells from the **S100A9-OE group** exhibited elevated ROS levels compared with coculture of cells from the **Vector group**. However, no significant difference in ROS levels was detected in HBMECs cocultured with A549 cells from the **S100A9-OE+si-NC group** and **S100A9-OE group**, although the ROS levels in HBMECs cocultured with A549 cells from the **S100A9-OE+si-Vimentin group** were significantly lower than those detected upon coculture with cells from the **S100A9-OE+si-NC group (Fig. 5M)**. These findings were corroborated with H1975 cell cocultures **(Sup Fig. 5M)**. These data indicate that S100A9 promotes ROS generation specifically via the upregulation of Vimentin expression.

Finally, the results of the Western blot analysis indicated that compared with HBMECs cocultured with cells from the **Vector group**, HBMECs cocultured with cells from the** S100A9-OE group** exhibited a significant decrease in VE-Cadherin expression and a marked increase in N-Cadherin and α-SMA expression. No significant differences in VE-Cadherin, N-Cadherin, or α-SMA levels were detected between the **S100A9-OE group** and the **S100A9-OE+si-NC group**. However, compared with HBMECs cocultured with cells from the **S100A9-OE+si-NC group**, HBMECs cocultured with cells from the **S100A9-OE+si-Vimentin group** exhibited significantly increased VE-Cadherin expression and significantly decreased N-Cadherin and α-SMA protein expression **(Fig. 5N-Q)**. Furthermore, comparable results were obtained with the H1975 systems **(Sup Fig. 5N-Q)**. These results demonstrate that S100A9 promotes EndMT in HBMECs through the upregulation of Vimentin expression.

### S100A9 Positively Regulates Vimentin Expression to Promote LUAD Metastasis and the Functional Impairment of HBMECs

**Figure 6:**
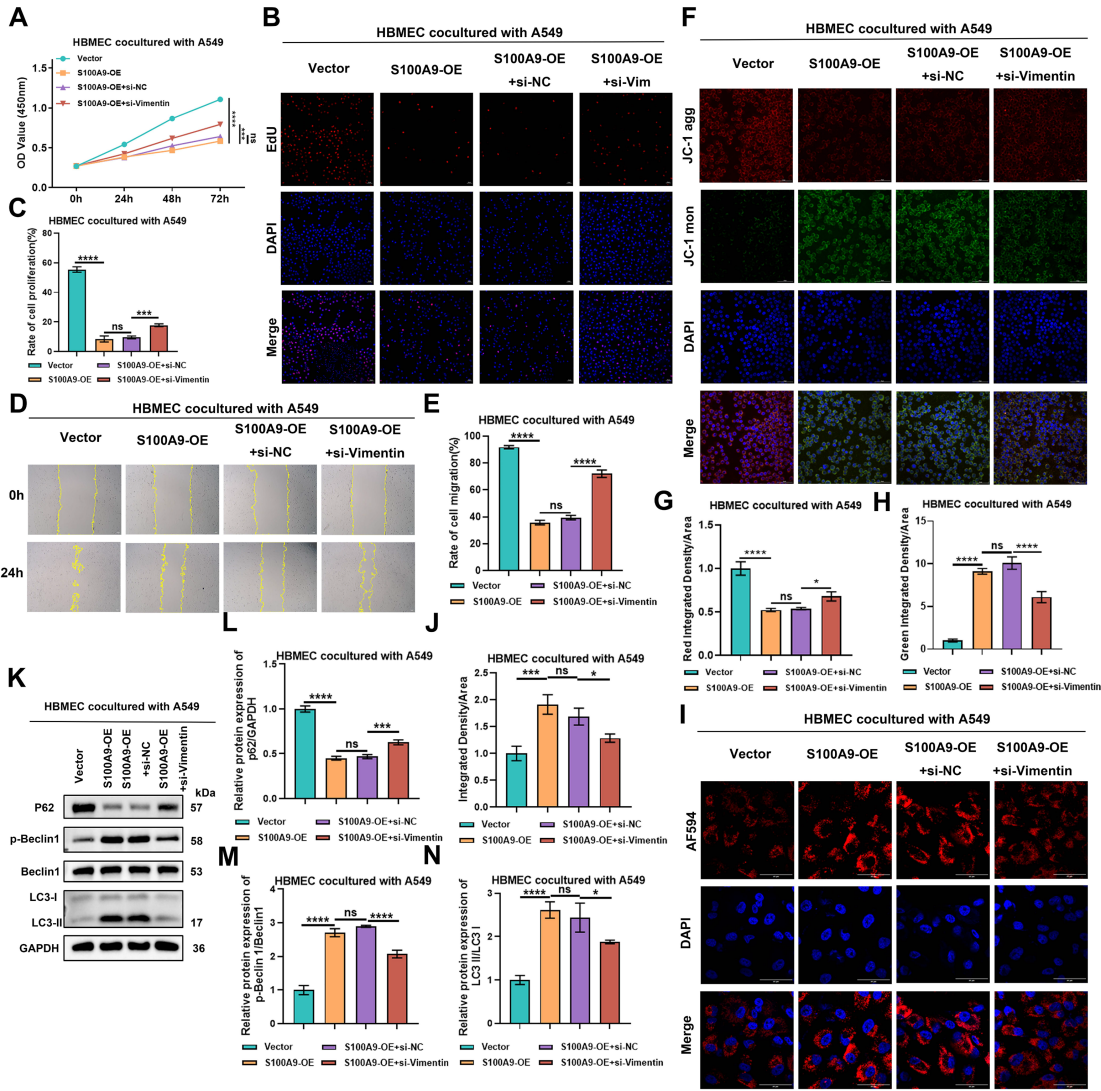
S100A9 positively regulates Vimentin expression to promote LUAD metastasis and increase HBMEC dysfunction. **Groups (A-N):** The following groups of cells were used in the experiments: Vector group A549 cells cocultured with HBMECs; S100A9-OE group A549 cells cocultured with HBMECs; S100A9-OE+si-NC group A549 cells cocultured with HBMECs; and S100A9-OE+si-Vimentin group A549 cells cocultured with HBMECs. **(A)** CCK-8 assay results showing OD values at 450 nm of HBMECs after 0, 24, 48, and 72 h of co-culture with A549 cells. ***P < 0.001; ****P < 0.0001; ns; n = 3 independent biological replicates. Data are presented as mean ± SD. **(B-C)** EdU assay results showing proliferation rates of HBMECs co-cultured with A549 cells. ***P < 0.001; ****P < 0.0001; ns; n = 3 independent biological replicates. Data are presented as mean ± SD. **(D-E)** Scratch assay results showing migration distances of HBMECs co-cultured with A549 cells. ****P < 0.0001; ns; n = 3 independent biological replicates. Data are presented as mean ± SD. **(F-H)** JC-1 assay results showing mitochondrial membrane potentials in HBMECs co-cultured with A549 cells. *P < 0.05; ****P < 0.0001; ns; n = 3 independent biological replicates. Data are presented as mean ± SD. **(I-J)** Confocal immunofluorescence results showing lysosomal pH in HBMECs co-cultured with A549 cells. *P < 0.05; ***P < 0.001; ns; n = 3 independent biological replicates. Data are presented as mean ± SD. **(K-N)** Western blot showing protein expression of P62, p-Beclin 1/Beclin 1, and LC3-II/I ratios in HBMECs co-cultured with A549 cells. *P < 0.05; ***P < 0.001; ****P < 0.0001; ns; n = 3 independent biological replicates. Data are presented as mean ± SD. Statistical significance was determined by one-way ANOVA followed by Tukey's post hoc test or two-way ANOVA followed by Bonferroni post hoc correction, as appropriate. Full-length blots/gels are presented in the raw image.

To elucidate the effects of S100A9 and Vimentin on the proliferation of HBMECs, cell counting kit-8 assays were conducted upon coculture with A549 cells. The optical density values of HBMECs cocultured with A549 cells from the **S100A9-OE group** at 0 h, 24 h, 48 h, and 72 h were significantly lower than those cocultured with A549 cells from the **Vector group**. No significant differences in the optical density values at 450 nm were observed at 0 h, 24 h, 48 h, or 72 h between the **S100A9-OE group** and the **S100A9-OE+si-NC group**. However, the optical density values at 450 nm at 0 h, 24 h, 48 h, and 72 h were significantly greater in the **S100A9-OE+si-Vimentin group** than in the** S100A9-OE+si-NC group (Fig. 6A)**. Similar trends were detected with H1975 cells **(Sup Fig. 6A)**, which suggested that S100A9 inhibits HBMEC proliferation mediated by increased Vimentin expression.

EdU assays demonstrated that compared with those in the **Vector group**, the proliferation rates of HBMECs cocultured with cells from the **S100A9-OE group** were significantly lower. However, the proliferation rates of HBMECs cocultured with cells from the **S100A9-OE+si-NC group** and **S100A9-OE group** did not significantly differ. Conversely, the proliferation rates of HBMECs cocultured with cells from the **S100A9-OE+si-Vimentin group** were significantly greater than those cultured with cells from the **S100A9-OE+si-NC group (Fig. 6B-C)**. Consistent data were obtained with H1975 cells **(Sup Fig. 6B-C)**, reinforcing the suppressive role of the S100A9/Vimentin axis on HBMEC proliferation.

Transwell migration assays were used to evaluate the migratory capacity of HBMECs. Upon coculture with A549 cells, the migration distance of HBMECs cocultured with cells from the **S100A9-OE group** was significantly lower than that of HBMECs cocultured with cells from the **Vector group**. No significant difference in migration distance was observed between HBMECs cocultured with cells from the **S100A9-OE+si-NC group** and the **S100A9-OE group**. However, the migration distance of HBMECs cocultured with cells from the **S100A9-OE+si-Vimentin group** was significantly greater than that of HBMECs cocultured with cells from the **S100A9-OE+si-NC group (Fig. 6D-E)**. Similar results were obtained with H1975 cells** (Sup Fig. 6D-E)**, indicating that S100A9 impedes HBMEC migration via a Vimentin-dependent mechanism.

The mitochondrial membrane potential of HBMECs was evaluated using JC-1 assays. In the context of A549 cells, HBMECs cocultured with cells from the **S100A9-OE group** presented decreased red fluorescence intensity and increased green fluorescence intensity compared with HBMECs cocultured with cells from the **Vector group**. No significant differences in red or green fluorescence intensity were observed between the **S100A9-OE group** and the **S100A9-OE+si-NC group**. However, HBMECs cocultured with cells from the **S100A9-OE+si-Vimentin group** displayed increased red fluorescence intensity and decreased green fluorescence intensity compared with HBMECs cocultured with cells from the **S100A9-OE+si-NC group (Fig. 6F-H)**. Similar results were obtained with H1975 cells** (Sup Fig. 6F-H),** suggesting that S100A9 disrupts the mitochondrial membrane potential via Vimentin signaling.

The lysosomal pH in HBMECs was assessed using specific pH-sensitive probes. In the context of A549 cells, HBMECs cocultured with cells from the **S100A9-OE group** exhibited a significantly decreased lysosomal pH and increased acidity compared with HBMECs cocultured with cells from the **Vector group**. No significant difference in lysosomal pH was observed between the **S100A9-OE group** and the **S100A9-OE+si-NC group**. Conversely, compared with the **S100A9-OE+si-NC group**, HBMECs cocultured with cells from the **S100A9-OE+si-Vimentin group** presented an increased lysosomal pH and reduced acidity** (Fig. 6I-J)**. Similar trends were detected with H1975 cells **(Sup Fig. 6I-J)**, indicating that S100A9 promotes lysosomal acidification via the upregulation of Vimentin expression.

Autophagic flux in HBMECs was evaluated through Western blot analysis. In the context of A549 cells, HBMECs cocultured with cells from the **S100A9-OE group** exhibited decreased P62 protein levels and increased ratios of p-Beclin 1/Beclin 1 and LC3-II/I protein expression compared with HBMECs cocultured with cells from the **Vector group**. No significant differences in P62 expression or the p-Beclin 1/Beclin 1 and LC3-II/I ratios were detected between the **S100A9-OE group** and the** S100A9-OE+si-NC group**. Conversely, compared with the **S100A9-OE+si-NC group**, HBMECs cocultured with cells from the **S100A9-OE+si-Vimentin group** displayed increased P62 expression and decreased ratios of p-Beclin 1/Beclin 1 and LC3-II/I protein expression **(Fig. 6K-N)**. Consistent results were obtained with H1975 cells **(Sup Fig. 6K-N)**, suggesting that S100A9 promotes autophagy induction through the upregulation of Vimentin expression.

### USP33 Modulates the Functions of HBMECs by Stabilizing the S100A9 Protein in PC-9 Cells

To identify proteins that interact with S100A9, co-IP coupled with mass spectrometry was conducted. A comprehensive list of the protein categories and their corresponding quantitative data is provided in **Table S2**.

**Figure 7:**
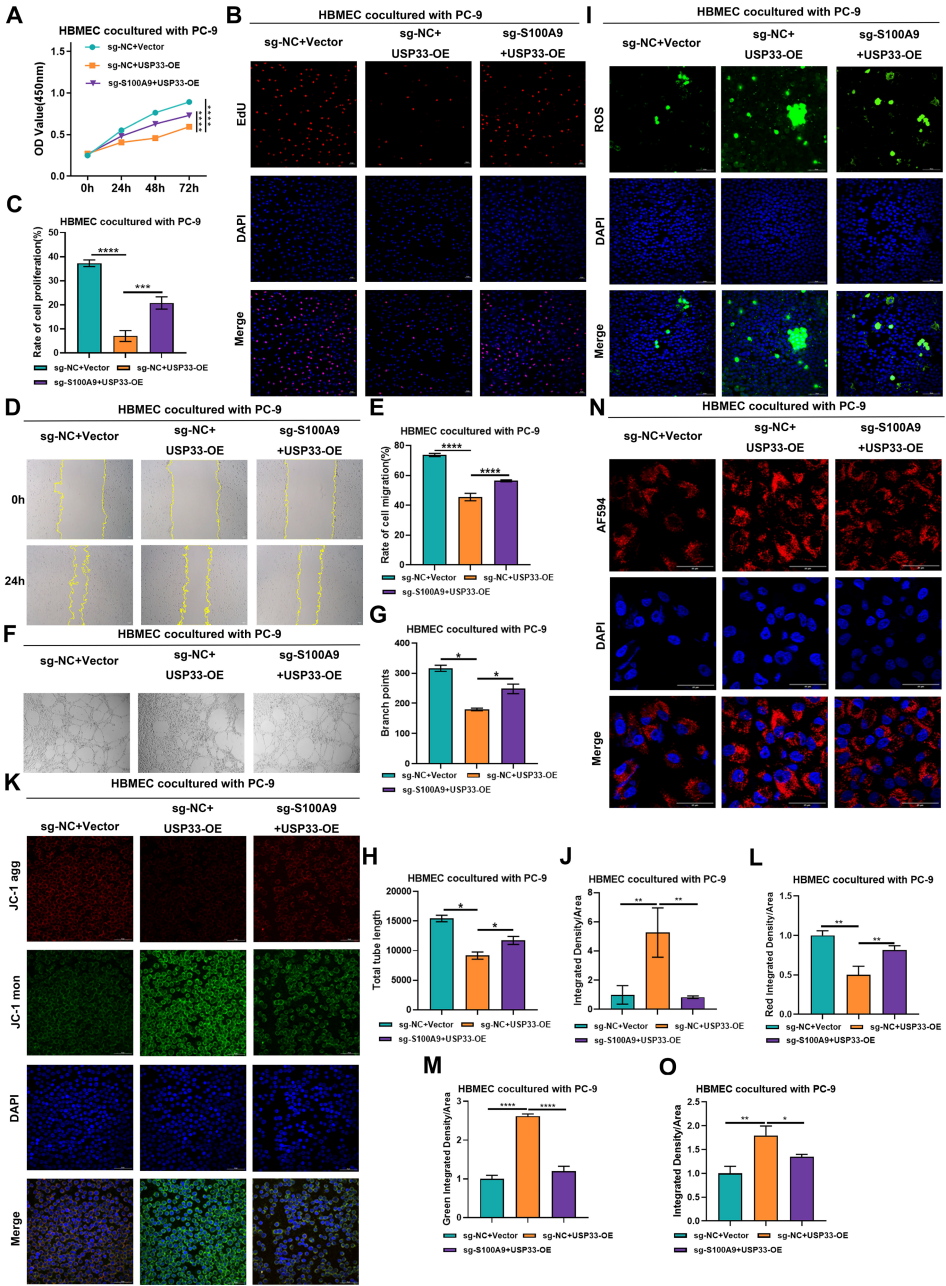
USP33 from PC-9 cells promotes the biological functions of HBMECs by stabilizing the expression of S100A9. **Groups (A-O):** HBMECs were co-cultured with PC-9 cells from one of the following groups: sg-NC+Vector, sg-NC+USP33-OE, or sg-S100A9+USP33-OE. **(A)** CCK-8 assay results showing OD values at 450 nm of HBMECs after 0, 24, 48, and 72 h of co-culture with PC-9 cells. ****P < 0.0001; n = 3 independent biological replicates. Data are presented as mean ± SD. **(B-C)** EdU assay results showing proliferation rates of HBMECs co-cultured with PC-9 cells. ***P < 0.001; ****P < 0.0001; n = 3 independent biological replicates. Data are presented as mean ± SD. **(D-E)** Scratch assay results showing migration distance of HBMECs co-cultured with PC-9 cells. ****P < 0.0001; n = 3 independent biological replicates. Data are presented as mean ± SD. **(F-H)** Tube formation assay results showing the number of branch points and tube lengths of HBMECs co-cultured with PC-9 cells. *P < 0.05; n = 3 independent biological replicates. Data are presented as mean ± SD. **(I-J)** ROS assay results showing reactive oxygen species levels in HBMECs co-cultured with PC-9 cells. ****P < 0.0001; n = 3 independent biological replicates. Data are presented as mean ± SD. **(K-M) J**C-1 assay results showing mitochondrial membrane potential in HBMECs co-cultured with PC-9 cells. ****P < 0.0001; n = 3 independent biological replicates. Data are presented as mean ± SD. **(N-O)** Confocal immunofluorescence staining results showing lysosomal pH in HBMECs co-cultured with PC-9 cells. ****P < 0.0001; n = 3 independent biological replicates. Data are presented as mean ± SD. Data were analyzed by one-way ANOVA followed by Tukey's post hoc test and two-way ANOVA followed by Bonferroni post hoc correction.

To elucidate the synergistic regulatory effect of USP33 and S100A9 on the proliferation of HBMECs, we used a coculture system that included PC-9 cells and performed CCK-8 assays. The results demonstrated that compared with those of HBMECs cocultured with cells from the **sg-NC+Vector group**, the OD450 values of the HBMECs in the **sg-NC+USP33-OE group** were significantly lower at 0, 24, 48, and 72 h. Notably, compared with those of HBMECs cocultured with cells from the **sg-NC+USP33-OE group**, the OD450 values of HBMECs cocultured with cells from the **sg-S100A9+USP33-OE group** increased **(Fig. 7A)**, suggesting that the USP33-mediated stabilization of S100A9 protein expression in LUAD cells inhibits HBMEC proliferation.

EdU assays revealed that the proliferation rate of HBMECs cocultured with cells from the **sg-NC+USP33-OE group** was significantly lower than that of HBMECs cocultured with cells from the **sg-NC+Vector group**. Conversely, the proliferation rate of HBMECs cocultured with cells from the **sg-S100A9+USP33-OE group** was significantly greater than that of HBMECs cocultured with cells from the **sg-NC+USP33-OE group (Fig. 7B-C)**, underscoring the role of USP33 in suppressing HBMEC proliferation through the upregulation of S100A9.

Scratch wound assays revealed that the migratory capacity of HBMECs cocultured with cells from the **sg-NC+USP33-OE group** was significantly lower than that of HBMECs cocultured with cells from the **sg-NC+Vector group**. In contrast, the migratory capacity of HBMECs cocultured with cells from the **sg-S100A9+USP33-OE group** was significantly greater than that of HBMECs cocultured with cells from the **sg-NC+USP33-OE group (Fig. 7D-E)**, suggesting that USP33 stabilizes S100A9 to inhibit HBMEC migration.

*In vitro* tube formation assays in which HBMECs were cocultured with PC-9 cells revealed that compared with HBMECs cocultured with cells from the **sg-NC+Vector group,** HBMECs cocultured with cells from the **sg-NC+USP33-OE group** exhibited significantly fewer branch points and shorter total lengths. Furthermore, compared with HBMECs cocultured with cells from the **sg-NC+USP33-OE group**, HBMECs cocultured with cells from the **sg-S100A9+USP33-OE group** displayed significantly more branch points and greater total lengths **(Fig. 7F-H)**, indicating that USP33 stabilizes S100A9 to suppress the angiogenesis of HBMECs.

Immunofluorescence analysis of ROS levels in HBMECs cocultured with PC-9 cells revealed that compared with HBMECs cocultured with cells from the **sg-NC+Vector group**, the ROS levels in HBMECs cocultured with cells from the** sg-NC+USP33-OE group** were significantly greater. In contrast, compared with HBMECs cocultured with cells from **the sg-NC+USP33-OE group**, the ROS levels in HBMECs cocultured with cells from** the sg-S100A9+USP33-OE** group were significantly lower **(Fig. 7I-J)**, suggesting that USP33 stabilizes S100A9 to promote ROS generation in HBMECs.

Assessment of mitochondrial membrane potential by JC-1 staining revealed that compared with HBMECs cocultured with PC-9 cells from the **sg-NC+Vector group**, the red fluorescence intensity in the **sg-NC+USP33-OE group** significantly decreased, and a concomitant increase in green fluorescence intensity was detected. Moreover, in contrast to the **sg-NC+USP33-OE group**, HBMECs cocultured with cells from the **sg-S100A9+USP33-OE group** exhibited a significant increase in red fluorescence intensity and a marked decrease in green fluorescence intensity **(Fig. 7K-M)**, demonstrating that USP33 stabilizes S100A9 to reduce the mitochondrial membrane potential in HBMECs.

The results of lysosomal pH measurements in HBMECs cocultured with PC-9 cells revealed that lysosomal acidity was significantly lower in HBMECs cocultured with cells from the **sg-NC+USP33-OE group** than in HBMECs cocultured with cells from the **sg-NC+Vector group**. Conversely, compared with HBMECs cocultured with cells from the **sg-NC+USP33-OE group,** HBMECs cocultured with cells from the **sg-S100A9+USP33-OE group** exhibited a significant increase in lysosomal acidity **(Fig. 7N-O)**, suggesting that USP33 stabilizes S100A9 to inhibit lysosomal acidification in HBMECs.

Collectively, these findings highlight the pivotal role of USP33 in modulating HBMEC proliferation, migration, angiogenesis, ROS generation, mitochondrial membrane potential, and lysosomal acidification through the stabilization of S100A9.

### USP33 Deubiquitinates S100A9-Ub-K48 to Stabilize S100A9 and Promote Vimentin Coexpression

**Figure 8:**
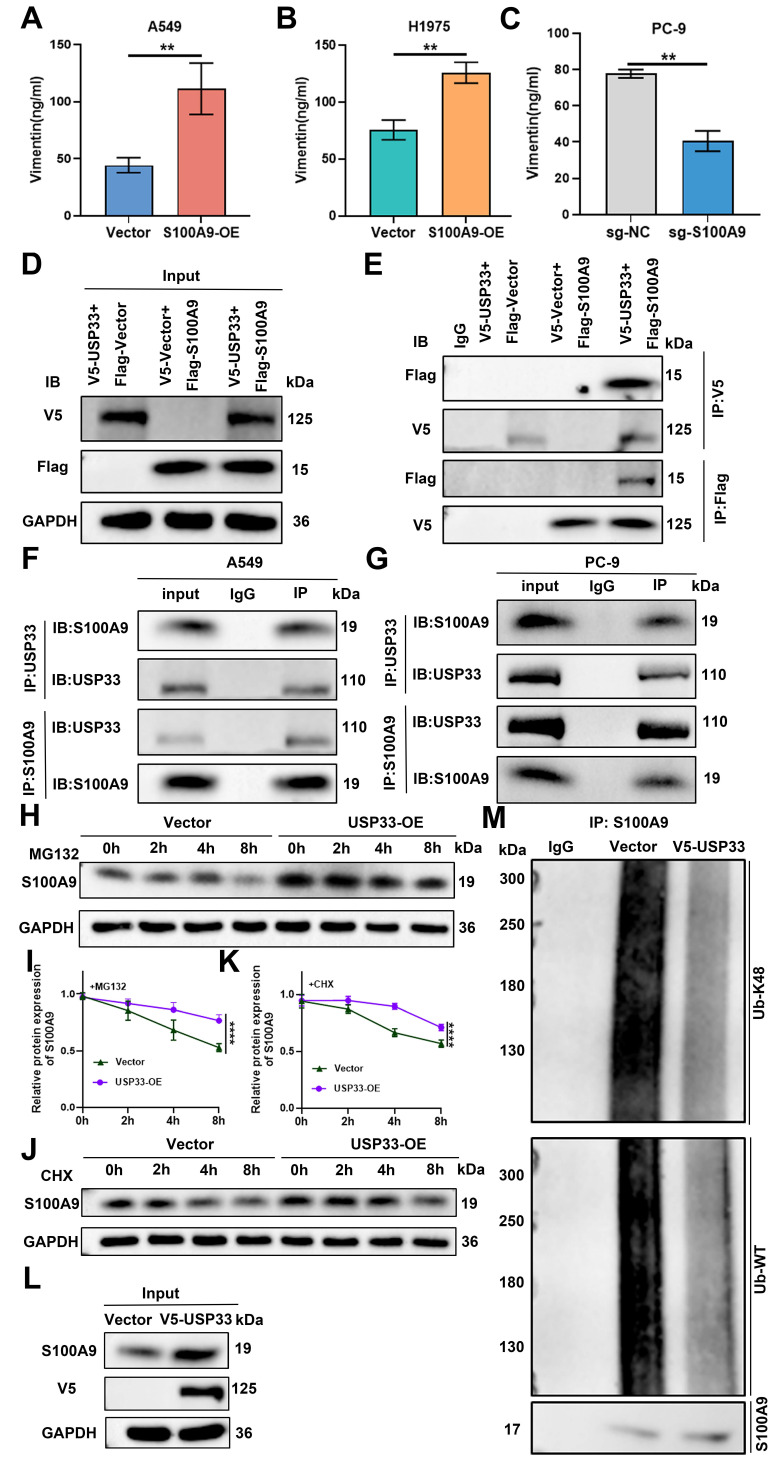
USP33 removes K48-linked polyubiquitin chains on S100A9 to stabilize its expression and promote Vimentin secretion. **(A-C)** ELISA results showing extracellular Vimentin secretion levels in A549 and H1975 cells from the Vector and S100A9-OE groups, and in PC-9 cells from the sg-NC and sg-S100A9 groups. **P < 0.01; n = 3 independent biological replicates. Data are presented as mean ± SD. **(D, E)** Co-IP assay results showing the interaction between USP33 and S100A9 when V5-tagged USP33 and Flag-tagged S100A9 were co-transfected into HEK293T cells. n = 3 independent biological replicates. Data are presented as mean ± SD. **(F, G)** Co-IP assay results showing the endogenous interaction between USP33 and S100A9 in A549 (F) and PC-9 (G) cells. n = 3 independent biological replicates. Data are presented as mean ± SD. **(H-K)** Western blot analysis showing S100A9 protein half-life in A549 cells from the Vector and USP33-OE groups after treatment with MG132 (H, I) or CHX (J, K). ****P < 0.0001; n = 3 independent biological replicates. Data are presented as mean ± SD. **(L-M)** IP results showing K48-linked polyubiquitination levels of S100A9 regulated by USP33 in Vector and USP33-OE A549 cells. n = 3 independent biological replicates. Data are presented as mean ± SD. Statistical significance was determined by Student's t test and two-way ANOVA followed by Bonferroni post hoc correction. Full-length blots/gels are presented in the raw image.

ELISA revealed that in A549 and H1975 cells, extracellular Vimentin secretion was significantly greater in the S100A9-OE groups than in the Vector groups. Consistent with these findings, in PC-9 cells, extracellular Vimentin secretion was significantly lower in the sg-S100A9 group than in the sg-NC group **(Fig. 8A-C)**. Coexpression of V5-tagged USP33-OE and Flag-tagged S100A9-OE constructs in HEK293T cells substantiated a robust association between these ectopically expressed proteins **(Fig. 8D-E)**. Additionally, the endogenous association between USP33 and S100A9 was confirmed in both A549 and PC-9 cells via co-IP **(Fig. 8F-G)**. CHX chase assays revealed that compared with Vector control cells, USP33-OE A549 cells displayed reduced protein degradation and a greater half-life throughout the monitored time course (0, 2, 4, and 8 h). This stabilization effect was observed in the presence of the proteasome inhibitor MG132 **(Fig. 8H-I)** and in the presence of CHX **(Fig. 8J-K)**. Subsequent deubiquitination assays revealed that USP33 overexpression significantly attenuated the K48-linked polyubiquitination of S100A9 compared with that of the Vector control **(Fig. 8L-M)**, indicating that USP33 stabilizes S100A9 by inhibiting its K48 ubiquitination.

## Discussion

LUAD remains the predominant driver of cancer-associated mortality worldwide and includes diverse demographic populations within China 39. The clinical outcomes of these patients are dictated primarily by local recurrence and distant metastasis, which serve as the main causes of death 40. Notably, in a French cohort receiving nivolumab therapy, the incidence of brain metastases was approximately 20%, indicating a robust correlation with advanced disease severity and progression 41. These intracranial metastatic lesions frequently cause substantial cognitive deficits, thereby severely compromising both quality of life and overall survival among various patient groups. Metastasis therefore remains a significant hurdle to therapeutic efficacy in lung cancer patients 42.

Existing clinical paradigms for managing LUAD-associated brain metastases include multimodal therapeutic strategies, such as radiotherapy, surgical resection, stereotactic radiosurgery, systemic chemotherapy, cancer stem cell-targeted therapies, and microRNA-based interventions. Despite these achievements, therapeutic efficacy remains limited by insufficient potency or undesirable toxicity, underscoring the urgent need to elucidate and characterize novel molecular targets 43. Previous single-cell transcriptomic profiling of primary tumors versus brain metastases revealed the marked enrichment of brain-metastatic adenocarcinoma cells within LUAD patients and suggested that S100A9 is a promising candidate biomarker 11. The vast majority of circulating tumor cells undergo rapid anoikis upon reaching the brain microenvironment 44. We previously revealed via multiplex immunohistochemistry that SNAI1-expressing LUAD cells can colonize discrete metastatic niches. This observation suggests that S100A9-positive cells exhibit greater potential to traverse the blood-brain barrier and colonize distinct cerebral regions.

In the present study, we further elucidated the functional roles of USP33 and extracellular Vimentin, an upstream regulatory deubiquitinase and a downstream effector of S100A9, respectively, in the intrinsic remodeling of the LUAD cellular landscape. Specifically, we reveal the molecular mechanisms whereby these genes undermine the structural and functional integrity of the blood-brain barrier, promoting its disruption and accelerating the pathogenesis of brain metastasis.

S100A9 encodes a calcium-binding protein belonging to the S100 family that frequently forms heterodimers with S100A8. This protein complex orchestrates multiple oncogenic effects, driving metastasis, tumor progression, and invasion while simultaneously establishing an immunosuppressive tumor microenvironment. In the context of metastatic breast cancer, Cowpea mosaic virus-mediated targeting of S100A9 has been demonstrated to recruit innate immune cells to pulmonary tissue, thereby attenuating lung metastasis 45. Furthermore, S100A9-conjugated viral vaccines have demonstrated therapeutic efficacy in mitigating pulmonary colonization in preclinical models of melanoma and triple-negative breast cancer 46.

LUAD brain metastasis represents a specific modality of distant dissemination. Notably, primary neuroblastomas also display metastatic potential mediated by elevated S100A9 expression 47. Consistent with previous data, the upregulation of S100A9 in LUAD cells compromises the integrity of the BBB. In the context of hepatitis B virus-associated hepatocellular carcinoma, NF-κB-induced S100A9 secretion drives neutrophil extracellular trap formation via the RAGE/TLR4-ROS axis, thereby facilitating tumor invasion and metastasis 48. Conversely, LUAD cells devoid of viral induction fail to display elevated S100A9 secretion but instead exhibit increased Vimentin release. The contribution of NETs to the pathophysiology of BBB disruption within this specific context remains unclear. Beyond its established role in metastatic progression, S100A9 confers chemoresistance in patients who present with brain metastases. Activation of the S100A9-RAGE-NF-κB-JUNB signaling axis constitutes a molecular hallmark of brain metastatic lesions spanning melanoma, lung, and breast adenocarcinomas. Consequently, S100A9 and its cognate receptor RAGE have emerged as promising biomarkers for liquid biopsy and as targets for radiosensitization 49, 50.

The results of the present study reveal that secreted S100A9 potentiates radioresistance across diverse malignancies via RAGE-mediated signaling, underscoring its critical function in brain colonization. However, the results of the integrated transcriptomic and proteomic binding assays in this study revealed that the secretion of extracellular vimentin precipitates BBB disruption. Furthermore, we identified USP33 as a deubiquitinase that stabilizes S100A9 via ubiquitin chain cleavage, thereby inhibiting its proteasomal degradation and potentiating its function in lung cancer brain metastasis.

Analysis of The Cancer Genome Atlas (TCGA) data confirmed marked S100A9 upregulation in LUAD tissues, which was significantly inversely correlated with patient prognosis. *In vitro* assays demonstrated that overexpression of S100A9 significantly increased cellular proliferation, migration, and invasion while concurrently attenuating apoptosis. *In vivo*, S100A9-overexpressing xenografts exhibited accelerated tumor growth and led to an increased metastatic burden. Moreover, left cardiac ventricle injection models corroborated the pivotal role of S100A9 in brain colonization, which is consistent with previous reports.

During LUAD pathogenesis, a premetastatic niche is established to facilitate tumor cell dissemination and survival 50. Breast cancer cells compromise endothelial integrity through calcium-dependent mechanisms and cytoskeletal reorganization 51. The results of the present study revealed that LUAD cells suppress HBMEC proliferation and tube formation, downregulate the expression of tight junction proteins (ZO-1, Occludin, and Claudin-5), and facilitate transendothelial migration. Elevated F-Na+ permeability was previously correlated with a decrease in transendothelial electrical resistance 52, 53. The results of the present study suggest that the detected increase in F-Na⁺ permeability may compromise monolayer integrity owing to osmotic stress. Upon TGF-β1 stimulation, primary rat brain endothelial cells undergo endothelial-mesenchymal transition, which is characterized by the depletion of junctional proteins and the acquisition of fibronectin expression 54. In conjunction with the results of previous investigations, the results of the present study reveal that elevated S100A9 expression facilitates EndMT, which may be mediated by the downregulation of tight junction proteins. Furthermore, elevated ROS levels in HBMECs are positively correlated with cytoskeletal disorganization and increased apoptosis activity 55.

The findings herein reveal that S100A9 in LUAD cells amplifies ROS accumulation in HBMECs. S100A9 functions as a pivotal signaling nexus and regulates the invasive-metastatic BBB disruption cascade in the context of LUAD via the USP33-S100A9-Vimentin axis, making it a key factor for prospective therapeutic intervention.

As a critical member of the ubiquitin-specific protease family, USP33 functions as a deubiquitinating enzyme and plays instrumental roles in the molecular pathogenesis of malignancies. Deubiquitinases counteract ubiquitination via the proteolytic cleavage of ubiquitin chains, thereby safeguarding the structural fidelity and stability of substrate proteins 56. USP33 increases the structural fidelity of 6-phosphofructo-2-kinase/fructose-2,6-biphosphatase 3 by inhibiting its ubiquitin-proteasomal degradation; consequently, the USP33-PFKFB3 axis potentiates aerobic glycolysis and accelerates the progression of multiple myeloma 57. Similarly, USP33 physically interacts with CTNNB1, inhibiting its proteasomal degradation and substantially increasing the proliferative capacity and stem-like properties of pancreatic cancer cells 58.

In the context of triple-negative breast cancer, the USP33-TAP63 axis suppresses tumor activity by precisely orchestrating autophagic and ferroptotic pathways 59. In the pancreatic cancer microenvironment, USP33 catalyzes the deubiquitination of p21-activated kinase 1, thereby attenuating its proteolytic turnover and conferring resistance to gemcitabine 60. Demonstrating exquisite substrate specificity, USP33 selectively targets K27- and K48-linked polyubiquitin chains at the K277 residue of CBX2, thereby increasing its protein stability and the proliferative and metastatic capacities of ovarian cancer cells 29. Moreover, USP33 enzymatically deubiquitinates transforming growth factor beta receptor 2 (TGFBR2), which reduces lysosomal catabolism and facilitates plasma membrane retention—key biological mechanisms that consolidate a self-sustaining proliferation-metastasis feedback loop in pancreatic neoplasms 30. Previous research has revealed that USP33 acts as a catalyst for esophageal cancer metastasis via the deubiquitination of integrin α6 61. In the gastric cancer milieu, USP33 physically interacts with roundabout guidance receptor 1 to modulate Slit2-Robo1 signaling by inhibiting Robo1 proteolytic degradation 62. Biochemically, ubiquitin moieties generate heterogeneous isopeptide linkages on specific lysine or N-terminal methionine residues 63. Notably, USP33 mitigates mitophagy-mediated apoptosis by cleaving K63-linked ubiquitin chains from mitochondrial substrates and exhibits a distinct enzymatic preference for K6-, K11-, K48-, and K63-linked polyubiquitin chains 64.

The findings of the current study reveal a novel regulatory axis governed by the USP33-S100A9-Vimentin cascade, which is pivotal for driving LUAD progression and compromising BBB integrity. While S100A9 has been correlated with tumoral inflammation and metastasis across diverse malignancies, its upstream regulatory mechanisms, specifically involving the modulation of deubiquitination, remain insufficiently characterized. Similarly, although Vimentin is a canonical EMT biomarker linked to metastatic phenotypes, its functional interaction with S100A9 within the context of LUAD and endothelial dysfunction remain insufficiently characterized. A pivotal finding of this investigation is the identification of USP33 as a deubiquitinase that directly engages with and stabilizes S100A9, which drives the transcriptional upregulation of Vimentin, thereby orchestrating the development of HBMECs with dysfunctional endothelial phenotypes.

We confirmed direct binding between USP33 and S100A9 via co-IP and detected their significant subcellular colocalization. USP33 preferentially hydrolyzes K48-linked polyubiquitin chains on S100A9, thereby preventing its proteasomal degradation, which corroborates the established enzymatic specificity of USP33. Cycloheximide chase assays and MG132 inhibition assays demonstrated that USP33 overexpression extends the half-life of S100A9 by slowing its degradation. Such molecular stabilization increases extracellular Vimentin expression and secretion, thereby potentiating the malignant phenotype of LUAD and exacerbating HBMEC injury.

In LUAD-HBMEC coculture systems, elevated USP33 expression increased S100A9 stability and potentiated the LUAD-mediated suppression of critical functions of HBMECs, including proliferation, migration, tube formation, and redox homeostasis. Through S100A9 signaling, USP33 increases extracellular Vimentin expression and secretion, compromises endothelial tight junction integrity, induces EndMT, disrupted mitochondrial membrane depolarization and cellular autophagy.

The Vimentin gene encodes a type III intermediate filament protein essential for the preservation of cytoskeletal structural integrity. The KPVY117L Vimentin mutant forms unit-length filaments but fails to polymerize into mature, functional networks, thereby effectively inhibiting cancer metastasis 65. The E3 ubiquitin ligase Neurl3 orchestrates Vimentin degradation, thereby repressing EMT and limiting nasopharyngeal carcinoma progression 66. Conversely, the long noncoding RNA LINC01559 sequesters Vimentin to block its ubiquitination and subsequent proteasomal degradation, thereby promoting the migration, invasion, and metastasis of LUAD cells 67. Although Vimentin ubiquitination has been reported, the molecular mechanisms governing its deubiquitination remain elusive. Our empirical data demonstrate that the expression of Vimentin is markedly upregulated by the synergistic regulation of USP33 and S100A9. Notably, phosphorylated Vimentin has been shown to attenuate stem cell-like metastasis and differentiation 68. Given the multifaceted posttranslational regulation of Vimentin, critical uncertainties, including whether ubiquitination and phosphorylation sites overlap or antagonize each other and whether specific E3 ligases facilitate Vimentin degradation, remain. Further rigorous investigation is warranted to determine whether USP33 indirectly modulates Vimentin deubiquitination via the intermediary S100A9. Given the distinct biological contributions of intracellular versus extracellular Vimentin, we hypothesize that the phenotypic manifestations are orchestrated predominantly by the secreted Vimentin fraction.

Transcriptomic analysis of A549 cells revealed that S100A9 overexpression led to the most significant upregulation of Vimentin mRNA expression. Functional assays validated the S100A9-mediated upregulation of Vimentin expression. In HBMECs cocultured with S100A9-overexpressing LUAD cells, Vimentin knockdown attenuated the increase in F-Na+ permeability. Moreover, the levels and fluorescence intensities of the tight junction proteins ZO-1, Occludin, and Claudin-5 were restored. Additionally, the silencing of vimentin expression abrogated the deficits in tube formation and ROS generation while ameliorating EndMT, as evidenced by the restored expression of VE-cadherin and decreased expression of N-cadherin and α-SMA. Vimentin knockdown rescued the proliferative and migratory impairments of HBMECs while attenuating lysosomal acidification and autophagic flux. These findings identify Vimentin as a pivotal downstream effector of S100A9, orchestrating tight junction compromise, EndMT, oxidative stress, and endothelial dysfunction through its increased expression and secretion, thereby facilitating LUAD brain metastasis.

During the invasive cascade, LUAD cells may release intracellular Vimentin into the systemic circulation, either directly or encapsulated within exosomes. This extracellular Vimentin directly engages specific receptors on the endothelial cell surface of the BBB. These interactions constitute a pathological insult upon brain microvascular endothelial cells within the BBB. Nevertheless, the precise mechanisms underlying this process remain incompletely understood and merit further investigation, which is a principal limitation of the current study.

Despite the deployment of multiple methodologies, including gain- and loss-of-function paradigms, coculture systems mimicking the BBB, transcriptomic profiling, IP-MS, and *in vivo* tumorigenesis and metastasis models, the experimental models cannot fully recapitulate the complex milieu *in vivo*, particularly the left ventricular injection model. Notably, this approach circumvents the natural metastatic cascade originating from primary lung tumorigenesis and local invasion, in which cells are directly introduced into the systemic circulation. Consequently, this model fails to comprehensively recapitulate organ-specific tropism or the cellular adaptations intrinsic to brain metastases, potentially constraining insights into the precise mechanisms of BBB transmigration.

## Conclusion

This research elucidates the pivotal mechanism through which the USP33-S100A9-Vimentin axis mediates LUAD brain metastasis. USP33 inhibits the proteasomal degradation of S100A9 and increases its stability via deubiquitination. Stabilized S100A9 increases extracellular Vimentin expression and secretion, thereby compromising BBB integrity, inducing EndMT and oxidative stress, and promoting HBMEC dysfunction to ultimately facilitate LUAD brain metastasis. Pharmacological modulation of the USP33-S100A9-Vimentin axis represents a promising therapeutic target to suppress LUAD brain metastases.

## Supplementary Material

Supplementary figures and tables.

## Data Availability

The data supporting the findings of this study are available from the corresponding author upon reasonable request.

## Abbreviations

ANOVA: Analysis of Variance
BBB: Blood-Brain Barrier
CCK-8: Cell Counting Kit-8
CHX: Cycloheximide
Co-IP: Co-Immunoprecipitation
DCFH-DA: 2',7'-Dichlorofluorescein Diacetate
EndMT: Endothelial-to-Mesenchymal Transition
FAK: Focal Adhesion Kinase
F-Na: Sodium Fluorescein
GO: Gene Ontology
GSEA: Gene Set Enrichment Analysis
HBMECs: Human Brain Microvascular Endothelial Cells
HGSC: High-Grade Serous Carcinoma
HRP: Horseradish Peroxidase
IP-MS: Immunoprecipitation-Mass Spectrometry
JC-1: 5,5′,6,6′-Tetrachloro-1,1′,3,3′-Tetraethylbenzimidazolylcarbocyanine Iodide
KEGG: Kyoto Encyclopedia of Genes and Genomes
LUAD: Lung Adenocarcinoma
NSCLC: Non-Small Cell Lung Cancer
OD: Optical Density
PBS: Phosphate-Buffered Saline
P/S: Penicillin‒Streptomycin
PT: Primary Tumor
PVDF: Polyvinylidene Fluoride
RIPA: Radioimmunoprecipitation Assay
ROS: Reactive Oxygen Species
sgRNA: Single-Guide RNA
SPF: Specific Pathogen-Free
TBST: Tris-Buffered Saline with Tween-20
TCGA: The Cancer Genome Atlas
TGF-β: Transforming Growth Factor Beta
Ub: Ubiquitin
USP33: Ubiquitin-Specific Peptidase 33
Vim: Vimentin

## References

[1] Sainz de Aja J, Dost AFM, Kim CF (2021). Alveolar progenitor cells and the origin of lung cancer. J Intern Med.

[2] Boire A, Brastianos PK, Garzia L, Valiente M (2020). Brain metastasis. Nat Rev Cancer.

[3] Sun Q, Xing P, Wang Q, Xia Z, Li J, Li Z (2025). ANKRD11 as a potential biomarker for brain metastasis from lung adenocarcinoma via cerebrospinal fluid liquid biopsy. Transl Lung Cancer Res.

[4] Dunne EG, Fick CN, Mastrogiacomo B, Tan KS, Toumbacaris N, Vanstraelen S (2024). Clinicopathologic and genomic features associated with brain metastasis after resection of lung adenocarcinoma. JTCVS Open.

[5] Sung H, Ferlay J, Siegel RL, Laversanne M, Soerjomataram I, Jemal A (2021). Global Cancer Statistics 2020: GLOBOCAN Estimates of Incidence and Mortality Worldwide for 36 Cancers in 185 Countries. CA Cancer J Clin.

[6] Bonert M, Schittenhelm J, Begum H, Lu JQ, Swaminath A, Juergens RA (2024). Neuroanatomical location of lung cancer brain metastases in 234 patients with a focus on cancer subtyping and biomarkers. PLoS One.

[7] Jin J, Cui Y, Niu H, Lin Y, Wu X, Qi X (2024). NSCLC Extracellular Vesicles Containing miR-374a-5p Promote Leptomeningeal Metastasis by Influencing Blood-Brain Barrier Permeability. Mol Cancer Res.

[8] Tang M, Liang K, Duan W, Xia S, Shi D, Li E (2024). Reactive astrocytes promote tumor progression by up-regulating tumor protocadherin 1 expression in lung cancer brain metastasis. Biochem Biophys Res Commun.

[9] Di Federico A, Hong L, Elkrief A, Thummalapalli R, Cooper AJ, Ricciuti B (2025). Lung adenocarcinomas with mucinous histology: clinical, genomic, and immune microenvironment characterization and outcomes to immunotherapy-based treatments and KRAS(G12C) inhibitors. Ann Oncol.

[10] Ren X, Zhang Y, Lyu Y, Jin B, Guo H, Wu J (2019). Lactate dehydrogenase and serum tumor markers for predicting metastatic status in geriatric patients with lung adenocarcinoma. Cancer Biomark.

[11] Wang Z, Wang Y, Chang M, Wang Y, Liu P, Wu J (2023). Single-cell transcriptomic analyses provide insights into the cellular origins and drivers of brain metastasis from lung adenocarcinoma. Neuro Oncol.

[12] Xu J, Lu W, Wei X, Zhang B, Yang H, Tu M (2024). Single-cell transcriptomics reveals the aggressive landscape of high-grade serous carcinoma and therapeutic targets in tumor microenvironment. Cancer Lett.

[13] Ma R, Zhou X, Zhai X, Wang C, Hu R, Chen Y (2024). Single-cell RNA sequencing reveals immune cell dysfunction in the peripheral blood of patients with highly aggressive gastric cancer. Cell Prolif.

[14] Chen Y, Wu Z, Yi X (2024). Elucidating the pan-oncologic landscape of S100A9: prognostic and therapeutic corollaries from an integrative bioinformatics and Mendelian randomization analysis. Sci Rep.

[15] Alewine C (2024). Macrophages Under the Influence of Tumor Mesothelin Weaken Host Defenses against Pancreatic Cancer Metastasis. Cancer Res.

[16] Chen JM, He J, Qiu JM, Yang GG, Wang D, Shen Z (2024). Netrin-1-CD146 and netrin-1-S100A9 are associated with early stage of lymph node metastasis in colorectal cancer. BMC Gastroenterol.

[17] Chen L, Shu P, Zhang X, Ye S, Tian L, Shen S (2024). S100A8-Mediated Inflammatory Signaling Drives Colorectal Cancer Progression via the CXCL5/CXCR2 Axis. J Cancer.

[18] Liu Y, Li M, Fang Z, Gao S, Cheng W, Duan Y (2024). Overexpressing S100A9 ameliorates NK cell dysfunction in estrogen receptor-positive breast cancer. Cancer Immunol Immunother.

[19] Feng J, Rouse CD, Coogan I, Byrd O, Nguyen E, Taylor L (2024). Regulation of Age-Related Lipid Metabolism in Ovarian Cancer. bioRxiv.

[20] Charan M, Mishra S, Shilo K, Zhang X, Verma AK, Ghanta P (2025). S100A9 enhances tumor immune suppression and cancer cell survival in small cell lung cancer. Cell Death Dis.

[21] Huang N, Tang J, Yi X, Zhang M, Li B, Cheng Y (2024). Glioma-derived S100A9 polarizes M2 microglia to inhibit CD8+T lymphocytes for immunosuppression via αvβ3 integrin/AKT1/TGFβ1. Biochim Biophys Acta Mol Cell Res.

[22] Hong WC, Lee DE, Kang HW, Kim MJ, Kim M, Kim JH (2023). CD74 Promotes a Pro-Inflammatory Tumor Microenvironment by Inducing S100A8 and S100A9 Secretion in Pancreatic Cancer. Int J Mol Sci.

[23] Özbay Kurt FG, Cicortas BA, Balzasch BM, De la Torre C, Ast V, Tavukcuoglu E (2024). S100A9 and HMGB1 orchestrate MDSC-mediated immunosuppression in melanoma through TLR4 signaling. J Immunother Cancer.

[24] Fan R, Satilmis H, Vandewalle N, Verheye E, Vlummens P, Maes A (2023). Tasquinimod suppresses tumor cell growth and bone resorption by targeting immunosuppressive myeloid cells and inhibiting c-MYC expression in multiple myeloma. J Immunother Cancer.

[25] Matas-Nadal C, Bech-Serra JJ, Gatius S, Gomez X, Ribes-Santolaria M, Guasch-Vallés M (2023). Biomarkers Found in the Tumor Interstitial Fluid may Help Explain the Differential Behavior Among Keratinocyte Carcinomas. Mol Cell Proteomics.

[26] Li P, Su G, Cui Y (2024). Integrative single-cell and bulk transcriptome analyses identify a distinct pro-tumor macrophage signature that has a major prognostic impact on glioblastomas. Clin Exp Med.

[27] Gao F, Feng Y, Hu X, Zhang X, Li T, Wang Y (2023). Neutrophils regulate tumor angiogenesis in oral squamous cell carcinoma and the role of Chemerin. Int Immunopharmacol.

[28] Deng L, Meng T, Chen L, Wei W, Wang P (2020). The role of ubiquitination in tumorigenesis and targeted drug discovery. Signal Transduct Target Ther.

[29] Chen J, Shan W, Jia Q, Chen Y, Jiang W, Tian Y (2024). USP33 facilitates the ovarian cancer progression via deubiquitinating and stabilizing CBX2. Oncogene.

[30] Liu X, Xu J, Shen B, Xu J, Jiang J (2023). USP33 promotes pancreatic cancer malignant phenotype through the regulation of TGFBR2/TGFβ signaling pathway. Cell Death Dis.

[31] Tabatabaee A, Nafari B, Farhang A, Hariri A, Khosravi A, Zarrabi A (2024). Targeting vimentin: a multifaceted approach to combatting cancer metastasis and drug resistance. Cancer Metastasis Rev.

[32] van Beijnum JR, Huijbers EJM, van Loon K, Blanas A, Akbari P, Roos A (2022). Extracellular vimentin mimics VEGF and is a target for anti-angiogenic immunotherapy. Nat Commun.

[33] Ren S, Han S, Wang L, Huang Y, Wu J, Wu G (2022). Minimally Invasive Surgery for ICH Evacuation Combined with Deferoxamine Treatment Increased Perihematomal Claudin-5 and ZO-1 Expression Levels and Decreased BBB Permeability in Rabbits. Front Neurol.

[34] Kim KA, Kim D, Kim JH, Shin YJ, Kim ES, Akram M (2020). Autophagy-mediated occludin degradation contributes to blood-brain barrier disruption during ischemia in bEnd.3 brain endothelial cells and rat ischemic stroke models. Fluids Barriers CNS.

[35] Zhang Y, Zhu W, Yu H, Yu J, Zhang M, Pan X (2019). P2Y4/TSP-1/TGF-β1/pSmad2/3 pathway contributes to acute generalized seizures induced by kainic acid. Brain Res Bull.

[36] Shuvalova M, Dmitrieva A, Belousov V, Nosov G The role of reactive oxygen species in the regulation of the blood-brain barrier. Tissue Barriers. 2024: 2361202.

[37] Tan Q, Yu D, Song L (2021). Atorvastatin disrupts primary human brain microvascular endothelial cell functions via prenylation-dependent mitochondrial inhibition and oxidative stress. Fundam Clin Pharmacol.

[38] Wu C, Yang M, Liu R, Hu H, Ji L, Zhang X (2020). Nicotine Reduces Human Brain Microvascular Endothelial Cell Response to Escherichia coli K1 Infection by Inhibiting Autophagy. Front Cell Infect Microbiol.

[39] Steeg PS, Camphausen KA, Smith QR (2011). Brain metastases as preventive and therapeutic targets. Nat Rev Cancer.

[40] Yuan X, Wang Z, Li C, Lv K, Tian G, Tang M (2022). Bacterial biomarkers capable of identifying recurrence or metastasis carry disease severity information for lung cancer. Front Microbiol.

[41] Barlesi F, Dixmier A, Debieuvre D, Raspaud C, Auliac JB, Benoit N (2025). Final 3-year results from the EVIDENS study, an observational study of nivolumab in non-small cell lung cancer. Oncoimmunology.

[42] Kim DW, Mehra R, Tan DSW, Felip E, Chow LQM, Camidge DR (2016). Activity and safety of ceritinib in patients with ALK-rearranged non-small-cell lung cancer (ASCEND-1): updated results from the multicentre, open-label, phase 1 trial. Lancet Oncol.

[43] Yousefi M, Bahrami T, Salmaninejad A, Nosrati R, Ghaffari P, Ghaffari SH (2017). Lung cancer-associated brain metastasis: Molecular mechanisms and therapeutic options. Cell Oncol (Dordr).

[44] Kienast Y, von Baumgarten L, Fuhrmann M, Klinkert WE, Goldbrunner R, Herms J (2010). Real-time imaging reveals the single steps of brain metastasis formation. Nat Med.

[45] Chung YH, Park J, Cai H, Steinmetz NF (2021). S100A9-Targeted Cowpea Mosaic Virus as a Prophylactic and Therapeutic Immunotherapy against Metastatic Breast Cancer and Melanoma. Adv Sci (Weinh).

[46] Chung YH, Ortega-Rivera OA, Volckaert BA, Jung E, Zhao Z, Steinmetz NF (2023). Viral nanoparticle vaccines against S100A9 reduce lung tumor seeding and metastasis. Proc Natl Acad Sci U S A.

[47] Chen X, Xue Y, Feng J, Tian Q, Zhang Y, Wang Q (2021). Identification S100A9 as a potential biomarker in neuroblastoma. Mol Biol Rep.

[48] Zhan X, Wu R, Kong XH, You Y, He K, Sun XY (2023). Elevated neutrophil extracellular traps by HBV-mediated S100A9-TLR4/RAGE-ROS cascade facilitate the growth and metastasis of hepatocellular carcinoma. Cancer Commun (Lond).

[49] Monteiro C, Miarka L, Perea-García M, Priego N, García-Gómez P, Álvaro-Espinosa L (2022). Stratification of radiosensitive brain metastases based on an actionable S100A9/RAGE resistance mechanism. Nat Med.

[50] Valiente M, Sepúlveda JM, Pérez A (2023). Emerging targets for cancer treatment: S100A9/RAGE. ESMO Open.

[51] Lee TH, Avraham HK, Jiang S, Avraham S (2003). Vascular endothelial growth factor modulates the transendothelial migration of MDA-MB-231 breast cancer cells through regulation of brain microvascular endothelial cell permeability. J Biol Chem.

[52] Nair AL, Groenendijk L, Overdevest R, Fowke TM, Annida R, Mocellin O (2023). Human BBB-on-a-chip reveals barrier disruption, endothelial inflammation, and T cell migration under neuroinflammatory conditions. Front Mol Neurosci.

[53] Seelbach M, Chen L, Powell A, Choi YJ, Zhang B, Hennig B (2010). Polychlorinated biphenyls disrupt blood-brain barrier integrity and promote brain metastasis formation. Environ Health Perspect.

[54] Krizbai IA, Gasparics Á, Nagyőszi P, Fazakas C, Molnár J, Wilhelm I (2015). Endothelial-mesenchymal transition of brain endothelial cells: possible role during metastatic extravasation. PLoS One.

[55] Yu Y, Wu Y, Wei J, Huang F, Mao F, Nong W (2022). NMDA mediates disruption of blood-brain barrier permeability via Rho/ROCK signaling pathway. Neurochem Int.

[56] Liu J, Cheng Y, Zheng M, Yuan B, Wang Z, Li X (2021). Targeting the ubiquitination/deubiquitination process to regulate immune checkpoint pathways. Signal Transduct Target Ther.

[57] Zhou B, Wang N, Chen Q, Ren J, Fu X, Cheng X (2023). Deubiquitinase USP33 promotes the glycolysis and growth of osteosarcoma by modifying PFKFB3 ubiquitination and degradation. Am J Cancer Res.

[58] Zhong B, Zhu Q, Wang L, Wang F, Zheng Y, Lin S (2023). USP33 enhances cell survival and stemness by deubiquitinating CTNNB1 in BXPC-3 and SW1990 cells. Cell Biol Int.

[59] Qu F, Jian W, Huang Y, Zhou X, Wang X, Li J (2025). Synergistic inhibition of TNBC by USP33 and TAP63 through autophagy and ferroptosis activation. Cell Mol Life Sci.

[60] He L, Li X, Zhen Y, He J, Wang C (2025). METTL3/IGF2BP3 axis promotes gemcitabine resistance of pancreatic cancer cells through regulating USP33-mediated PAK1 deubiquitination and degradation. Naunyn Schmiedebergs Arch Pharmacol.

[61] Hang Q, Zuo S, Yang Y, Wang Y, Li C, Li W (2024). USP33 is an integrin α6 deubiquitinase and promotes esophageal squamous cell carcinoma cell migration and metastasis. J Cancer Res Clin Oncol.

[62] Xia Y, Wang L, Xu Z, Kong R, Wang F, Yin K (2019). Reduced USP33 expression in gastric cancer decreases inhibitory effects of Slit2-Robo1 signalling on cell migration and EMT. Cell Prolif.

[63] Iwai K, Fujita H, Sasaki Y (2014). Linear ubiquitin chains: NF-κB signalling, cell death and beyond. Nat Rev Mol Cell Biol.

[64] Niu K, Fang H, Chen Z, Zhu Y, Tan Q, Wei D (2020). USP33 deubiquitinates PRKN/parkin and antagonizes its role in mitophagy. Autophagy.

[65] Berr AL, Wiese K, Dos Santos G, Koch CM, Anekalla KR, Kidd M (2023). Vimentin is required for tumor progression and metastasis in a mouse model of non-small cell lung cancer. Oncogene.

[66] Zhou SQ, Feng P, Ye ML, Huang SY, He SW, Zhu XH (2024). The E3 ligase NEURL3 suppresses epithelial-mesenchymal transition and metastasis in nasopharyngeal carcinoma by promoting vimentin degradation. J Exp Clin Cancer Res.

[67] Feng H, Xu D, Jiang C, Chen Y, Wang J, Ren Z (2024). LINC01559 promotes lung adenocarcinoma metastasis by disrupting the ubiquitination of vimentin. Biomark Res.

[68] Kuburich NA, den Hollander P, Castaneda M, Pietilä M, Tang X, Batra H (2023). Stabilizing vimentin phosphorylation inhibits stem-like cell properties and metastasis of hybrid epithelial/mesenchymal carcinomas. Cell Rep.

