# Supplementary material for: USP33 Promotes Lung Adenocarcinoma Brain Metastasis by Inhibiting the K48-Linked Ubiquitination and Degradation of S100A9 and Facilitating Vimentin Secretion
Source: Int J Biol Sci. 2026 May 29;22(11):5852–77. doi: 10.7150/ijbs.127941 (PMC13282746; doi:10.7150/ijbs.127941)
Supplement: Supplementary file 1 — Supplementary figures and tables. [file ijbsv22p5852s1.zip › Supplementary Materials.pdf]

**Supplementary Figure 1.** Effects of S100A9 overexpression on the malignancy of A549 and H1975 cells.

**Groups of A549 and H1975 cells:** The Vector group and S100A9-OE group were used in the following experiments.

**(A-B)** Results of the CCK8 assay showing the OD values at 450 nm of A549 or H1975 cells at 0 h, 24 h, 48 h, and 72 h; ****P < 0.0001; N=3 independent biological replicates. The data are the mean ± SD. **(C-F)** Single-colony formation assay results showing the number of single clones formed by A549 or H1975 cells; ****P < 0.0001; N=3 independent biological replicates. The data are the mean ± SD. **(G-J)** Results of the EdU assay showing the proliferation rates of A549 and H1975 cells; ****P < 0.0001; N=3 independent biological replicates. The data are the mean ± SD. **(K-N)** Transwell assay results showing the number of migrating A549 or H1975 cells. ****P < 0.0001; N=3 independent biological replicates. The data are the mean ± SD. Statistical significance was determined using Student's t test and two-way ANOVA followed by the Bonferroni post hoc correction.

**Supplementary Figure 2.** Effects of S100A9 overexpression on the malignancy of A549 and H1975 cells.

**Groups of A549 and H1975 cells:** The Vector group and S100A9-OE group were used in the following experiments.

**(A-D)** Transwell assay results showing the number of invasive A549 or H1975 cells; ****P < 0.0001; N=3 independent biological replicates. The data are the mean ± SD. **(E-H)** Scratch assay results showing the widths of the migration distances of A549 and H1975 cells at 0 h and 24 h; ***P < 0.001; ****P < 0.0001; N=3 independent biological replicates. The data are the mean ± SD. **(I-L)** TUNEL assay results showing the percentages of apoptotic of A549 and H1975 cells. ****P < 0.0001; N=3 independent biological replicates. The data are the mean ± SD. Statistical significance was determined using Student's t test followed by the Bonferroni post hoc correction.

**Supplementary Figure 3.** Effects of S100A9 knockout on the malignancy of PC-9 cells.

**Groups (A–M):** The sg-NC group, sg-S100A9-1 group, and sg-S100A9-2 group were used in the following experiments.

**(A)** The results of the CCK8 assay showing the OD values at 450 nm of PC-9 cells at 0 h, 24 h, 48 h, and 72 h; ****P < 0.0001; N=3 independent biological replicates. The data are the mean ± SD. **(B-C)** The results of the EdU assay showing the cell proliferation rates of PC-9 cells; ****P < 0.0001; N=3 independent biological replicates. The data are the mean ± SD. **(D-E)** The single-colony formation assay results showing the number of single colonies formed by PC-9 cells; ****P < 0.0001; N=3 independent biological replicates. The data are the mean ± SD. **(F-G)** Scratch assay results showing the widths of the migration distances of PC-9 cells at 0 h and 24 h. **(H-K)** Numbers of migrating cells and invasive PC-9 cells in the Transwell assay; ****P < 0.0001; N=3 independent biological replicates. The data are the mean ± SD. **(L-M)** TUNEL assay results showing the percentage of apoptotic PC-9 cells. ****P < 0.0001; N=3 independent biological replicates. The data are the mean ± SD. Statistical significance was determined using one-way ANOVA followed by Tukey's post hoc test.

**Supplementary Figure 4:** H1975 cells: The Vector group and S100A9-OE group were used in the following experiments.

**(A)** The results of the CCK8 assay showing the OD values at 450 nm of HBMECs after 0 h, 24 h, 48 h, and 72 h of coculture with H1975 cells. ****P < 0.0001; N=3 independent biological replicates. The data are the mean ± SD. **(B)** The sodium ion permeability assay showing the sodium ion permeability of HBMECs cocultured with H1975 cells. **P < 0.01; N=3 independent biological replicates. The data are the mean ± SD. **(C-F)** Western blot assay results showing the expression levels of the osmotic pressure-maintaining proteins ZO-1, Occludin, and Claudin-5 in HBMECs cocultured with H1975 cells. **P<0.01; ****P<0.0001; N=3 independent biological replicates. The data are the mean ± SD. **(G-I)** Tube formation assay results showing the number of branches and the total lengths of the tubes formed by HBMECs cocultured with H1975 cells. *P<0.05; **P<0.01; N=3 independent biological replicates. The data are the mean ± SD. Statistical significance was determined using one-way ANOVA followed by

Tukey's post hoc test. **(J-K)** Cell viability of A549 and H1975 cells was assessed via the CCK-8 assay across the Vector group, S100A9-OE group, and S100A9-OE+ITGB1 inhibitor group. ****P<0.01; N=3 independent biological replicates. The data are the mean ± SD. **(L-Q)** Transwell assays were utilized to quantify the migratory and invasive capacities of A549 and H1975 cells in the Vector group, S100A9-OE group, and S100A9-OE+ITGB1 inhibitor group. ****P<0.01; N=3 independent biological replicates. The data are the mean ± SD.

**Supplementary Figure 5:**

**Groups (A–Q):** The following groups of cells were used in the experiments: Vector group H1975 cells cocultured with HBMECs; S100A9-OE group H1975 cells cocultured with HBMECs; S100A9-OE+si-NC group H1975 cells cocultured with HBMECs; and S100A9-OE+si-Vimentin group H1975 cells cocultured with HBMECs.

**(A-C)** RT–qPCR and Western blot results showing the RNA and protein expression levels, respectively, of Vimentin in H1975 cells transfected with si-1, si-2, and si-3. **P<0.01; ***P<0.001; ****P<0.0001; N=3 independent biological replicates. The data are the mean ± SD. **(D)** The results of the sodium ion permeability experiment showing the sodium ion permeability of HBMECs cocultured with H1975 cells. **P<0.01; ****P<0.0001; N=3 independent biological replicates. The data are the mean ± SD. **(E)** Immunofluorescence staining results showing the fluorescence intensities of ZO-1, Claudin-5, and Occludin in HBMECs cocultured with H1975 cells. N=3 independent biological replicates. The data are the mean ± SD. **(F-I)** Western blot experiment results showing the protein expression levels of ZO-1, Claudin-5, and Occludin in HBMECs cocultured with H1975 cells. ***P<0.001; ****P<0.0001; N=3 independent biological replicates. The data are the mean ± SD. **(J-L)** Tube formation experiment results showing the number of branch points and tube lengths of HBMECs cocultured with H1975 cells. ***P<0.001; ****P<0.0001; N=3 independent biological replicates. The data are the mean ± SD. **(M)** Reactive oxygen species (ROS) experiment results showing the reactive oxygen species content in HBMECs cocultured with H1975 cells. N=3 independent biological replicates. The data are the mean ± SD. **(N-**

**Q)** Western blot showing the protein expression levels of VE-Cadherin, N-Cadherin, and α-SMA in HBMECs cocultured with H1975 cells. ***P<0.001; ****P<0.0001; N=3 independent biological replicates. The data are the mean ± SD. Statistical analysis was performed using one-way analysis of variance (ANOVA).

**Supplementary Figure 6:**

**Groups (A–N):** The following groups of cells were used in the experiments: Vector group H1975 cells cocultured with HBMECs; S100A9-OE group H1975 cells cocultured with HBMECs; S100A9-OE+si-NC group H1975 cells cocultured with HBMECs; and S100A9-OE+si-Vimentin group H1975 cells cocultured with HBMECs.

**(A)** Results of the CCK8 assay showing the OD values at 450 nm of HBMECs after 0 h, 24 h, 48 h, and 72 h of coculture with H1975 cells; ***P<0.001; ****P<0.0001; N=3 independent biological replicates. The data are the mean ± SD. **(B-C)** Results of the EdU assay showing the proliferation rates of HBMECs cocultured with H1975 cells; ***P<0.001; ****P<0.0001; N=3 independent biological replicates. The data are the mean ± SD. **(D-E)** Scratch assay results showing the migration distance of HBMECs cocultured with H1975 cells; ***P<0.001; ****P<0.0001; N=3 independent biological replicates. The data are the mean ± SD. **(F-H)** Results of the JC-1 assay showing the mitochondrial membrane potential in of HBMECs cocultured with H1975 cells; N=3 independent biological replicates. The data are the mean ± SD. **(I-J)** Immunofluorescence confocal microscopy results showing the pH of lysosomes in HBMECs cocultured with H1975 cells; N=3 independent biological replicates. The data are the mean ± SD. **(K-N)** Western blot results of the protein expression level of the autophagy-related protein p62 and the p-Beclin 1/Beclin 1 and LC3-II/I ratios in HBMECs cocultured with H1975 cells. *P<0.05; **P<0.01; ****P<0.0001; N=3 independent biological replicates. The data are the mean ± SD. Statistical analysis was performed using one-way analysis of variance (ANOVA).

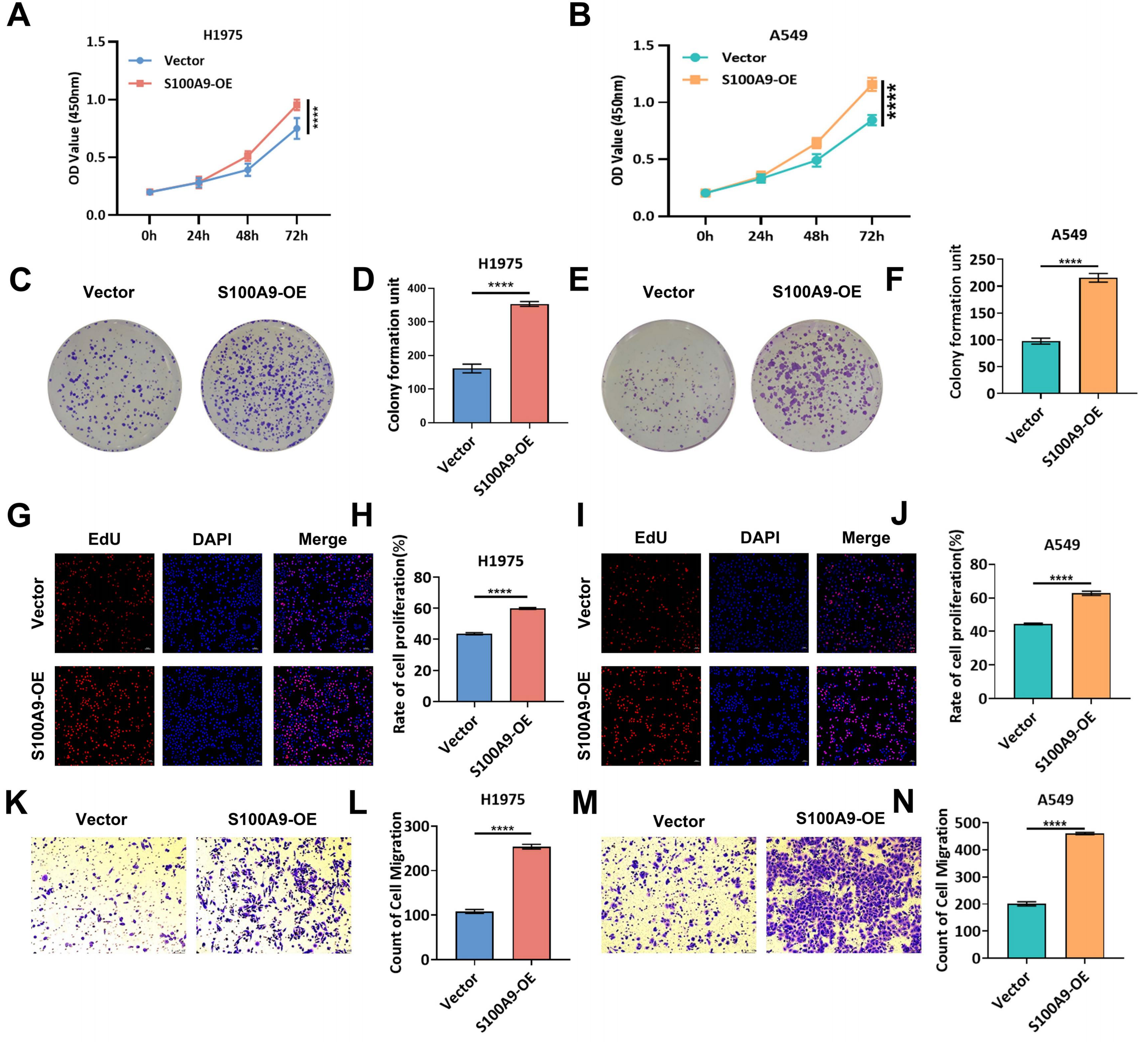

A
H1975
Vector
S100A9-OE
OD Value (450nm)
1.5
1.0
0.5
0.0
0h
24h
48h
72h
****
B
A549
Vector
S100A9-OE
OD Value (450nm)
1.5
1.0
0.5
0.0
0h
24h
48h
72h
****
C
Vector
S100A9-OE
D
H1975
Colony formation unit
400
300
200
100
0
****
Vector
S100A9-OE
E
Vector
S100A9-OE
F
A549
Colony formation unit
250
200
150
100
50
0
****
Vector
S100A9-OE
G
EdU
DAPI
Merge
Vector
S100A9-OE
H
H1975
Rate of cell proliferation(%)
80
60
40
20
0
****
Vector
S100A9-OE
I
EdU
DAPI
Merge
Vector
S100A9-OE
J
A549
Rate of cell proliferation(%)
80
60
40
20
0
****
Vector
S100A9-OE
K
Vector
S100A9-OE
L
H1975
Count of Cell Migration
300
200
100
0
****
Vector
S100A9-OE
M
Vector
S100A9-OE
N
A549
Count of Cell Migration
500
400
300
200
100
0
****
Vector
S100A9-OE

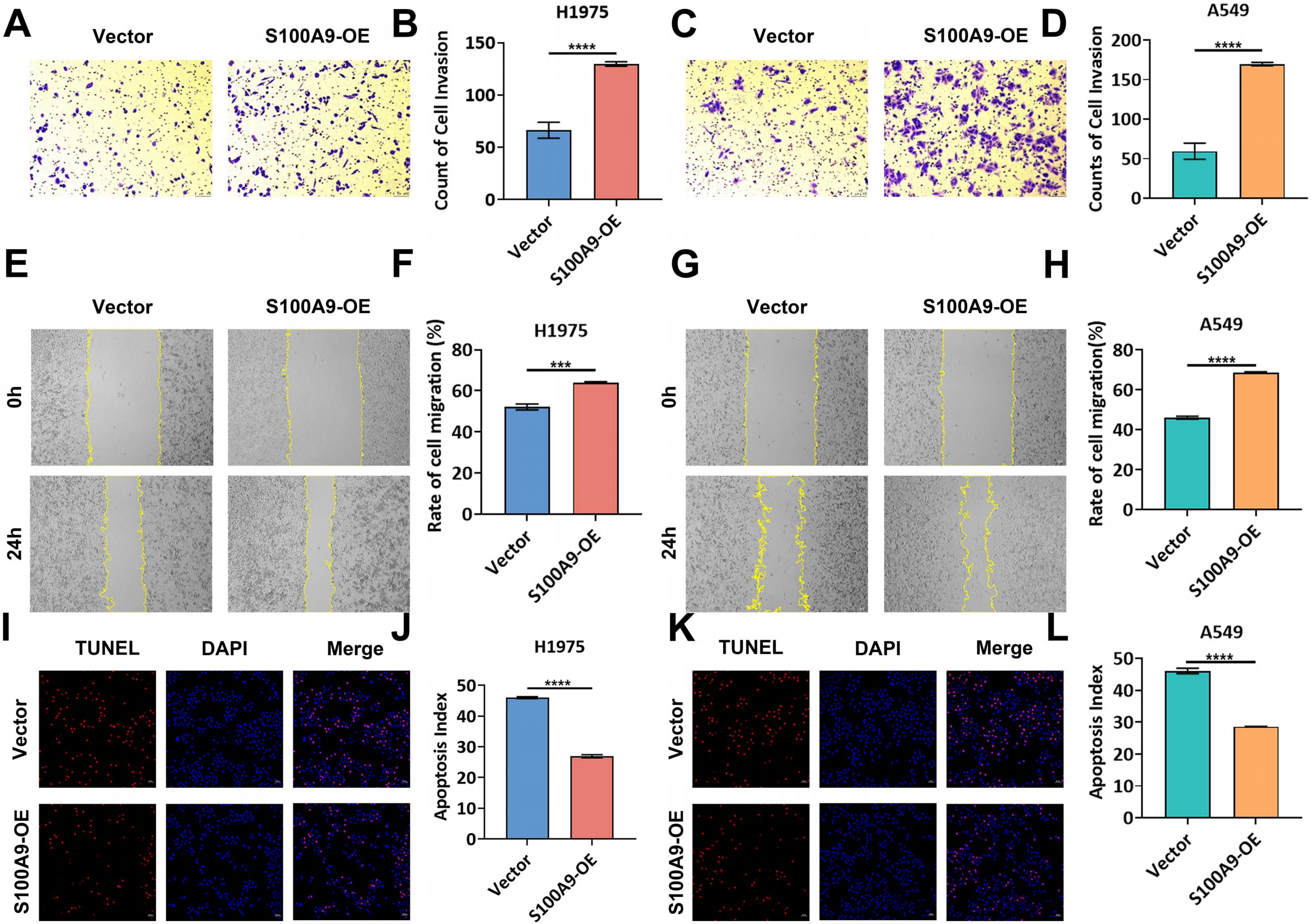
A
Vector
S100A9-OE
B
H1975
Count of Cell Invasion
150
100
50
0
****
Vector
S100A9-OE
C
Vector
S100A9-OE
D
A549
Counts of Cell Invasion
200
150
100
50
0
****
Vector
S100A9-OE
E
Vector
S100A9-OE
0h
24h
F
H1975
Rate of cell migration (%)
80
60
40
20
0
***
Vector
S100A9-OE
G
Vector
S100A9-OE
0h
24h
H
A549
Rate of cell migration(%)
80
60
40
20
0
****
Vector
S100A9-OE
I
TUNEL
DAPI
Merge
Vector
S100A9-OE
J
H1975
Apoptosis Index
50
40
30
20
10
0
****
Vector
S100A9-OE
K
TUNEL
DAPI
Merge
Vector
S100A9-OE
L
A549
Apoptosis Index
50
40
30
20
10
0
****
Vector
S100A9-OE

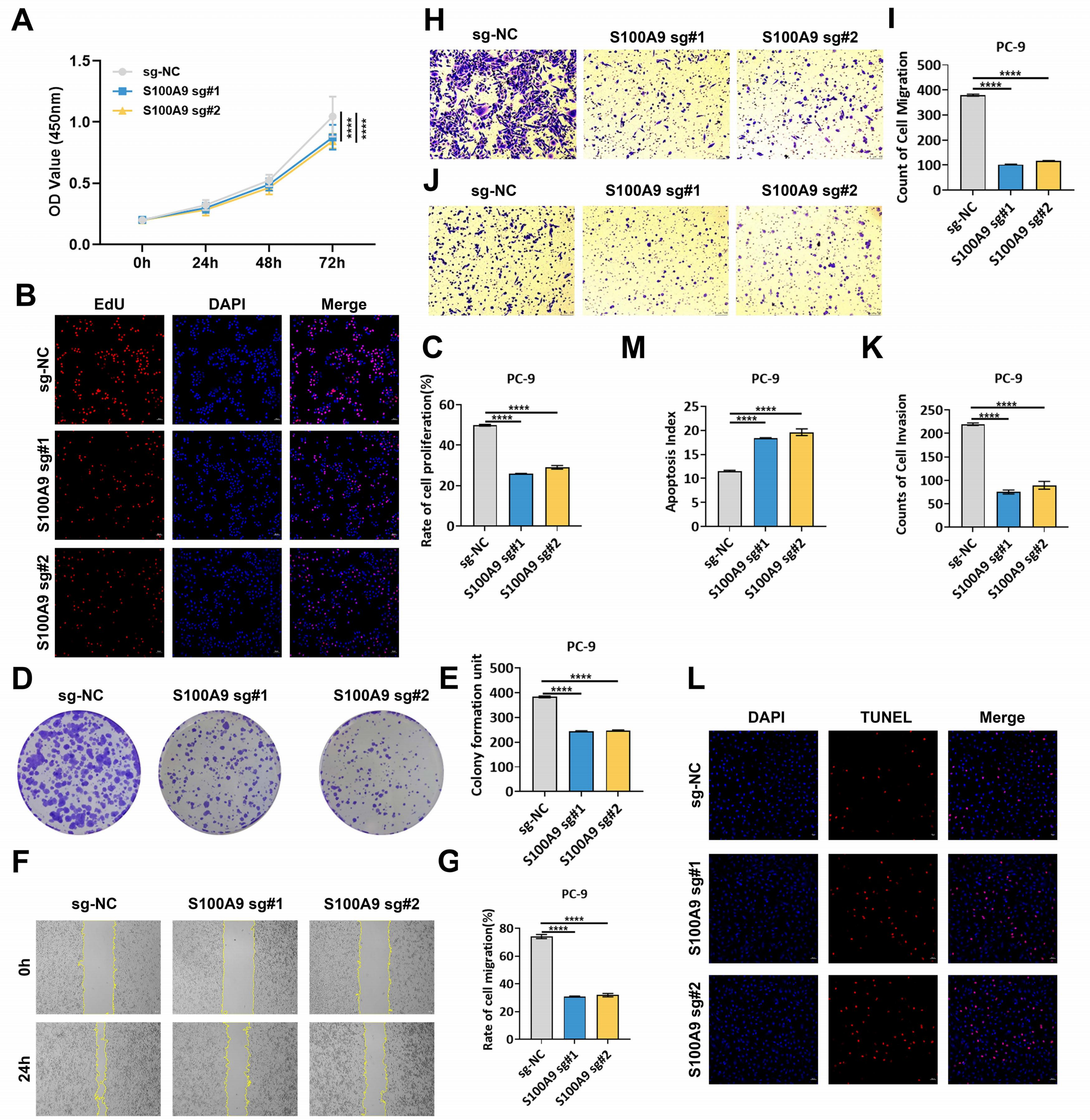
A
sg-NC
S100A9 sg#1
S100A9 sg#2
OD Value (450nm)
0.0
0.5
1.0
1.5
0h
24h
48h
72h
****
****
B
EdU
DAPI
Merge
sg-NC
S100A9 sg#1
S100A9 sg#2
C
PC-9
Rate of cell proliferation(%)
****
****
D
sg-NC
S100A9 sg#1
S100A9 sg#2
E
PC-9
Colony formation unit
F
sg-NC
S100A9 sg#1
S100A9 sg#2
0h
24h
G
PC-9
Rate of cell migration(%)
H
sg-NC
S100A9 sg#1
S100A9 sg#2
I
PC-9
Count of Cell Migration
J
sg-NC
S100A9 sg#1
S100A9 sg#2
K
PC-9
Counts of Cell Invasion
L
DAPI
TUNEL
Merge
sg-NC
S100A9 sg#1
S100A9 sg#2
M
PC-9
Apoptosis Index

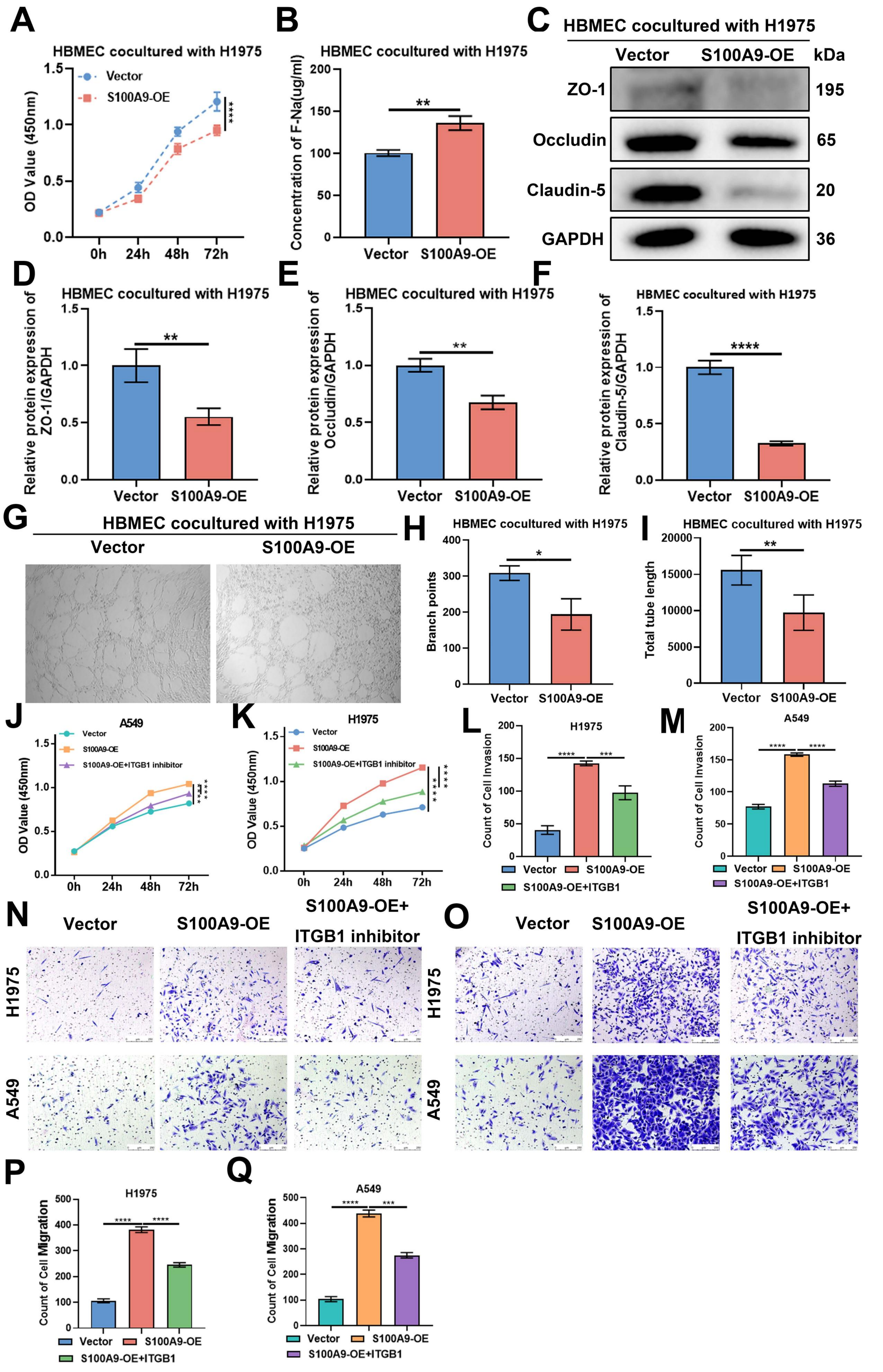
A
HBMEC cocultured with H1975
Vector
S100A9-OE
OD Value (450nm)
0h
24h
48h
72h
****
B
HBMEC cocultured with H1975
Concentration of F-Na(ug/ml)
**
Vector
S100A9-OE
C
HBMEC cocultured with H1975
Vector
S100A9-OE
kDa
ZO-1
195
Occludin
65
Claudin-5
20
GAPDH
36
D
HBMEC cocultured with H1975
Relative protein expression of ZO-1/GAPDH
**
E
HBMEC cocultured with H1975
Relative protein expression of Occludin/GAPDH
**
F
HBMEC cocultured with H1975
Relative protein expression of Claudin-5/GAPDH
****
G
HBMEC cocultured with H1975
Vector
S100A9-OE
H
HBMEC cocultured with H1975
Branch points
*
I
HBMEC cocultured with H1975
Total tube length
**
J
A549
Vector
S100A9-OE
S100A9-OE+ITGB1 inhibitor
OD Value (450nm)
K
H1975
Vector
S100A9-OE
S100A9-OE+ITGB1 inhibitor
OD Value (450nm)
L
H1975
Count of Cell Invasion
****
***
Vector
S100A9-OE
S100A9-OE+ITGB1
M
A549
Count of Cell Invasion
****
****
Vector
S100A9-OE
S100A9-OE+ITGB1
N
Vector
S100A9-OE
S100A9-OE+
ITGB1 inhibitor
H1975
A549
O
Vector
S100A9-OE
S100A9-OE+
ITGB1 inhibitor
H1975
A549
P
H1975
Count of Cell Migration
****
****
Vector
S100A9-OE
S100A9-OE+ITGB1
Q
A549
Count of Cell Migration
****
***
Vector
S100A9-OE
S100A9-OE+ITGB1

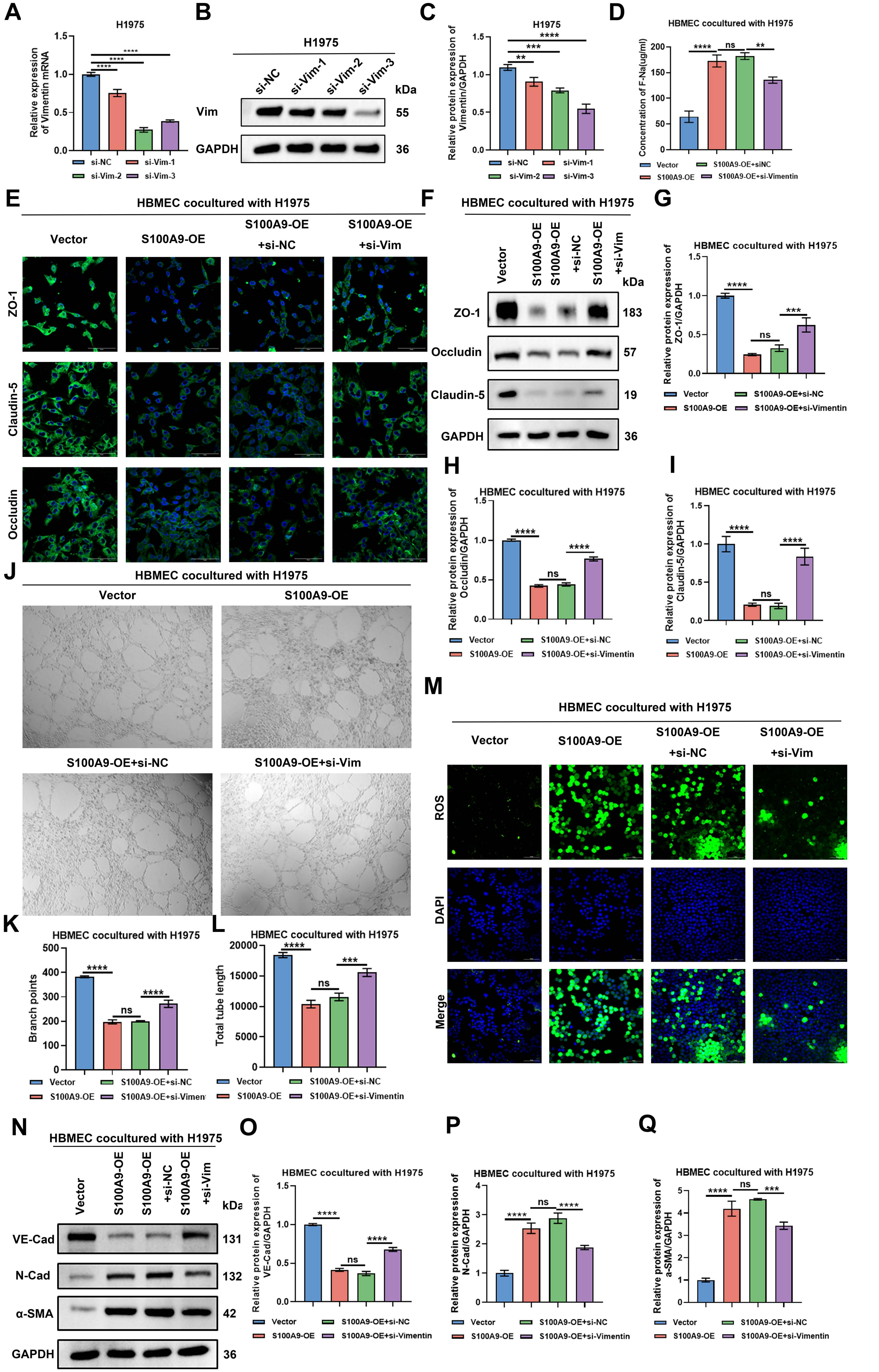

A
H1975
Relative expression of Vimentin mRNA
si-NC
si-Vim-1
si-Vim-2
si-Vim-3
B
H1975
si-NC si-Vim-1 si-Vim-2 si-Vim-3
kDa
Vim 55
GAPDH 36
C
H1975
Relative protein expression of Vimentin/GAPDH
D
HBMEC cocultured with H1975
Concentration of F-Na(ug/ml)
Vector
S100A9-OE
S100A9-OE+siNC
S100A9-OE+si-Vimentin
E
HBMEC cocultured with H1975
Vector
S100A9-OE
S100A9-OE +si-NC
S100A9-OE +si-Vim
ZO-1
Claudin-5
Occludin
F
HBMEC cocultured with H1975
Vector
S100A9-OE
S100A9-OE +si-NC
S100A9-OE +si-Vim
kDa
ZO-1 183
Occludin 57
Claudin-5 19
GAPDH 36
G
HBMEC cocultured with H1975
Relative protein expression of ZO-1/GAPDH
H
HBMEC cocultured with H1975
Relative protein expression of Occludin/GAPDH
I
HBMEC cocultured with H1975
Relative protein expression of Claudin-5/GAPDH
J
HBMEC cocultured with H1975
Vector
S100A9-OE
S100A9-OE+si-NC
S100A9-OE+si-Vim
K
HBMEC cocultured with H1975
Branch points
L
HBMEC cocultured with H1975
Total tube length
M
HBMEC cocultured with H1975
Vector
S100A9-OE
S100A9-OE +si-NC
S100A9-OE +si-Vim
ROS
DAPI
Merge
N
HBMEC cocultured with H1975
Vector
S100A9-OE
S100A9-OE +si-NC
S100A9-OE +si-Vim
kDa
VE-Cad 131
N-Cad 132
α-SMA 42
GAPDH 36
O
HBMEC cocultured with H1975
Relative protein expression of VE-Cad/GAPDH
P
HBMEC cocultured with H1975
Relative protein expression of N-Cad/GAPDH
Q
HBMEC cocultured with H1975
Relative protein expression of a-SMA/GAPDH
Vector
S100A9-OE
S100A9-OE+si-NC
S100A9-OE+si-Vimentin

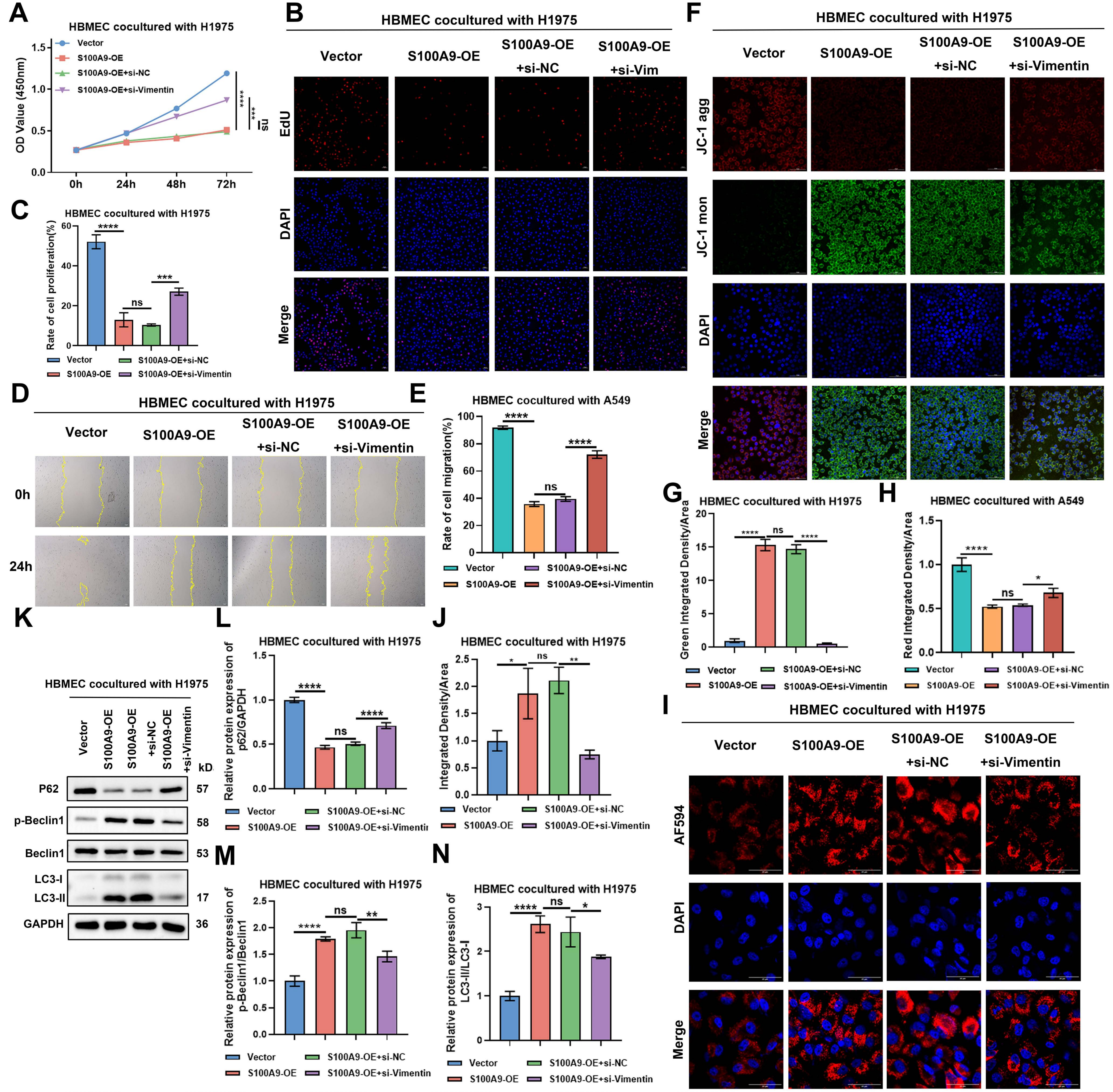
A
HBMEC cocultured with H1975
Vector
S100A9-OE
S100A9-OE+si-NC
S100A9-OE+si-Vimentin
OD Value (450nm)
0h
24h
48h
72h
****
***
ns
B
HBMEC cocultured with H1975
Vector
S100A9-OE
S100A9-OE +si-NC
S100A9-OE +si-Vim
EdU
DAPI
Merge
C
HBMEC cocultured with H1975
Rate of cell proliferation(%)
D
HBMEC cocultured with H1975
Vector
S100A9-OE
S100A9-OE +si-NC
S100A9-OE +si-Vimentin
0h
24h
E
HBMEC cocultured with A549
Rate of cell migration(%)
F
HBMEC cocultured with H1975
JC-1 agg
JC-1 mon
DAPI
Merge
G
HBMEC cocultured with H1975
Green Integrated Density/Area
H
HBMEC cocultured with A549
Red Integrated Density/Area
I
HBMEC cocultured with H1975
AF594
DAPI
Merge
K
HBMEC cocultured with H1975
Vector
S100A9-OE
S100A9-OE +si-NC
S100A9-OE +si-Vimentin
kD
P62 57
p-Beclin1 58
Beclin1 53
LC3-I
LC3-II 17
GAPDH 36
L
HBMEC cocultured with H1975
Relative protein expression of p62/GAPDH
J
HBMEC cocultured with H1975
Integrated Density/Area
M
HBMEC cocultured with H1975
Relative protein expression of p-Beclin1/Beclin1
N
HBMEC cocultured with H1975
Relative protein expression of LC3-II/LC3-I

**Table 1. Primer for following genes**

| Gene name | Primer sequence |
|---|---|
| S100A9 Forward | GGTCATAGAACACATCATGGAGG |
| S100A9 Reverse | GGCCTGGCTTATGGTGGTG |
| GAPDH Forward | GGAGCGAGATCCCTCCAAAAT |
| GAPDH Reverse | GGCTGTTGTCATACTTCTCATGG |
| Vimentin Forward | AGTCCACTGAGTACCGGAGAC |
| Vimentin Reverse | CATTTCACGCATCTGGCGTTC |
